# Serological Protection After Routine Measles-Containing Vaccination Among Children Aged 1–5 Years in Low- and Middle-Income Countries: A Systematic Review

**DOI:** 10.3390/vaccines14090760

**Published:** 2026-08-31

**Authors:** Imtiaz Hussain, Babar Tasneem Shaikh, Haider Abbas, Ahmad Khan, Sajid Bashir Soofi

**Affiliations:** 1 School of Public Health, Health Services Academy, Islamabad 44000, Pakistan; shaikh.babar@gmail.com (B.T.S.); haiderjamal12@gmail.com (H.A.); 2 Center of Excellence in Women and Child Health, Aga Khan University, Karachi 74800, Pakistan; ahmad.sherali@aku.edu (A.K.); sajid.soofi@aku.edu (S.B.S.); 3 Department of Paediatrics and Child Health, Aga Khan University, Karachi 74800, Pakistan

**Keywords:** measles, measles-containing vaccine (MCV), seroprotection, routine immunization, low- and middle-income countries (LMICs), systematic review

## Abstract

**Background**: Measles remains a major cause of morbidity and mortality among children in low- and middle-income countries (LMICs), despite widespread implementation of routine two-dose measles-containing vaccine (MCV) schedules. Serological assessment provides an objective measure of vaccine-induced immunity; however, evidence describing post-vaccination serological protection among children in LMICs remains fragmented and methodologically heterogeneous. **Objective**: To synthesize evidence on serological protection following routine two-dose MCV among children aged 1–5 years in LMICs and to identify determinants of immunity, methodological limitations, and evidence gaps relevant to immunization programmes. **Methods**: This systematic review was conducted in accordance with the Preferred Reporting Items for Systematic Reviews and Meta-Analyses (PRISMA 2020) guidelines and prospectively registered in PROSPERO (CRD420261300545). Literature searches were conducted in PubMed, MEDLINE, Scopus, Cochrane Library, CINAHL (EBSCO), and SciSpace on 21 January 2026. Two reviewers independently screened studies, extracted data, and assessed quality of evidence using Joanna Briggs Institute critical appraisal tools. Substantial clinical and methodological heterogeneity precluded meta-analysis; therefore, findings were synthesized narratively. **Results**: Eighteen studies met the eligibility. These studies were from 14 LMICs across four WHO regions (61.1% African Region, 27.8% Eastern Mediterranean Region, 5.6% each South-East Asia and the region of the Americas). Reported MCV seroprotection following completion of the two-dose schedule generally ranged from 30.6% to 100%, with the highest levels (87–100%), but with evident lower protection in specific settings, such as rural Pakistan (30.6%) and Ethiopia (40–54%) despite having documented vaccination records. Lower protection was consistently associated with undernutrition, human immunodeficiency virus (HIV) infection, incomplete or delayed vaccination, younger age at first dose, and weak programme performance. Considerable variability was observed in study design, vaccination schedules, laboratory assays (ELISA, PRNT, haemagglutination inhibition, and others), seroprotective thresholds, timing of antibody assessment, and participant characteristics. Risk of bias was largely moderate-to-high, and certainty of evidence ranged from low to very low. **Conclusions**: Routine two-dose measles vaccination generally provides substantial serological protection among children aged 1–5 years in LMICs. Nevertheless, methodological heterogeneity and geographical evidence gaps limit direct comparisons and reduce confidence in cross-study interpretation. Greater standardization of serological assessment and thresholds, harmonized reporting methods, and well-designed longitudinal studies are needed to strengthen the evidence base and support immunization policies aimed at achieving and sustaining measles elimination.

## 1. Introduction

Despite the availability of a safe, inexpensive, and effective vaccine for more than five decades, measles remains one of the leading causes of vaccine-preventable morbidity and mortality among children, particularly in low- and middle-income countries (LMICs) [1,2]. Measles virus is among the most contagious human pathogens, with its basic reproduction number (R_0_) commonly cited as 12 to 18, enabling rapid transmission in susceptible populations [1,3]. Although routine immunization programmes and supplementary immunization activities (SIAs) have substantially reduced measles mortality, a recent resurgence in many regions reflects persistent immunity gaps caused by declining vaccination coverage, health service disruptions, population displacement, and inequitable access to immunization services [4,5]. In 2023, an estimated 10.3 million measles cases and approximately 107,500 measles-associated deaths occurred globally, with the majority of deaths occurring among children younger than five years of age [5,6]. This burden of measles remains concentrated in LMICs, where weak health systems, population mobility, humanitarian emergencies, conflict, and shortages of healthcare workers contribute to inequitable access to routine immunization services, leading to persistent immunity gaps [7,8,9]. Interruptions to routine immunization during the COVID-19 pandemic further increased the number of zero-dose and under-immunized children, reversing years of progress toward measles elimination in many settings [10,11]. Because sustained interruption of measles transmission requires approximately 95% population immunity, even a small cluster of susceptible population can lead to a large outbreak [3,6,12].

Such immunity gaps are not merely theoretical but have major public health consequences. They are already contributing to major measles outbreaks in South Asia. In Pakistan, 4541 laboratory-confirmed cases were reported during the first quarter of 2026, followed by 71 measles-related deaths among children by the end of April [13]. Earlier evidence from Karachi similarly showed that although nearly 90% of children were reported to have received at least one measles vaccine dose, only 54% had detectable measles IgG antibodies [14]. Bangladesh experienced a simultaneous nationwide outbreak, with 19,161 suspected cases, 2973 laboratory-confirmed cases, and 30 confirmed deaths reported between 15 March and 14 April 2026 [15,16]. These outbreaks demonstrate that reported vaccination coverage may conceal substantial population susceptibility and underscore the importance of serological evidence for identifying residual immunity gaps.

Routine immunization with two appropriately timed doses of measles-containing vaccine (MCV), determined by national epidemiology and immunization schedules, remains the cornerstone of global measles prevention strategies [6]. In countries with ongoing measles transmission and risk of infant mortality due to measles, the first dose is commonly administered at 9 months of age, followed by the second dose according to the national schedule. The second dose provides an additional opportunity for seroconversion among children who fail to develop protective immunity following the first dose, rather than merely boosting immunity among children who have already responded to the first dose [1,17]. However, vaccine-induced immunity may differ according to age at vaccination, persistence of maternally derived antibodies, immune maturity, nutritional status, and underlying immunocompromising conditions [18,19].

Administrative vaccination coverage is the most commonly used indicator of immunization programme performance; however, it does not directly measure whether vaccinated children have developed protective immunity [20,21]. Coverage estimates reflect that vaccine delivery may overestimate population immunity when vaccination is delayed, documentation is incomplete, vaccine potency is compromised, or host-related factors impair seroconversion [6,22]. Serological assessment provides a direct measure of measles-specific antibodies and can identify immunity gaps that may not be apparent from administrative coverage data alone [2,20,23]. Nevertheless, serological testing is not regularly recommended as a way of confirming immunity status following receipt of age-appropriate MCV. In routine immunization practice, documented evidence of age-appropriate MCV is generally considered adequate proof of presumptive protection. Serological assessment can only be useful as an additional epidemiological tool to assess population-level immunity status, investigate suspected immunity gaps, and evaluate discrepancies between documented vaccination coverage and measurable antibody protection.

Protective immunity following measles vaccination is generally assessed by measuring measles-specific immunoglobulin G (IgG) antibodies or functional virus-neutralizing antibodies using laboratory assays such as enzyme-linked immunosorbent assays (ELISA), enzyme immunoassays (EIA), plaque reduction neutralization tests (PRNT), haemagglutination inhibition assays, particle agglutination assays, or more recently, multiplex bead-based immunoassays [24,25]. PRNT is widely regarded as the laboratory reference standard because it directly quantifies virus-neutralizing antibodies, whereas ELISA-based assays remain the most frequently used in epidemiological studies because of their operational simplicity and scalability [22,23,26,27]. Nevertheless, interpretation of serological findings remains challenging because of differences in laboratory assays, calibration standards, timing of blood sampling, and antibody cut-offs for defining protection. Although a neutralizing antibody concentration of approximately 120 mIU/mL is commonly cited as a correlate of protection against clinical measles, this threshold is derived primarily from historical challenge and outbreak studies and cannot be directly extrapolated across all commercial immunoassays [28,29]. Consequently, reported seroprotection may vary substantially between studies because of methodological differences rather than true biological variation in immunity.

Despite growing interest in measles serosurveillance, evidence on serological protection following routine doses of measles vaccination among under-five children in LMICS remains fragmented. Previous systematic reviews have primarily focused on vaccine effectiveness, vaccination before 9 months of age, maternal antibody interference, measles epidemiology, or methodological aspects of serosurveillance rather than synthesizing evidence on serological protection following routine vaccination in children aged 1–5 years [18,19,20]. Moreover, published studies substantially differ in design, assay type, seroprotection thresholds, vaccination verification methods, timing of assessment, participant age, and analytical approaches, making direct comparison difficult across studies [6,23]. Consequently, despite an increasing number of individual serological investigations, there remains limited consolidated evidence describing the extent of serological protection achieved following routine measles vaccination in LMICs or explaining the biological, programmatic, and methodological factors that contribute to variation in reported immunity.

This evidence gap is particularly important because children younger than five years continue to account for the greatest burden of measles-associated morbidity and mortality globally and remain the principal target population of the national Expanded Programme on Immunization (EPI) [5,6]. Reliable estimates of vaccine-induced serological protection are essential for evaluating immunization programme performance, identifying susceptible populations, informing supplementary immunization activities, and supporting evidence-based vaccination policy. Furthermore, understanding the determinants of Serological protection has become increasingly relevant as many countries transition from mortality reduction to regional measles elimination, where identifying relatively small immunity gaps may have substantial implications for outbreak prevention and for maintaining elimination status [2,4]. In this context, systematic synthesis of serological evidence can provide valuable insights beyond those obtainable from vaccination coverage or routine surveillance data alone.

This systematic review synthesized evidence on serological protection following routine measles vaccination among children aged 1–5 years in LMICs. Specifically, it examines reported levels of serological protection, biological and programmatic determinants of immunity, methodological heterogeneity across studies, and evidence gaps relevant to future serosurveillance and immunization policy.

## 2. Materials and Methods

**Study design:** This study was conducted and reported in accordance with the Preferred Reporting Items for Systematic Reviews and Meta-Analyses (PRISMA 2020) guidance (Table S1) and was guided by a prospectively registered protocol (PROSPERO: CRD420261300545, registered on 4 February 2026) [30]. The protocol initially proposed a meta-analysis where appropriate. However, after full-text review and data extraction, substantial clinical and methodological heterogeneity across study designs, vaccination schedules, laboratory methods, seroprotection thresholds, and outcome reporting precluded meaningful quantitative pooling. Findings were therefore synthesized using a structured narrative approach consistent with PRISMA 2020 and Cochrane guidelines [30,31].

**Eligibility criteria:** Eligible studies included children aged 1–5 years residing in LMICs who had received measles-containing vaccination and for whom laboratory-confirmed measles serological outcomes were reported. Studies including broader pediatric age groups were also considered eligible only when relevant subgroup data for children aged 1–5 years were extractable or when the study population substantially overlapped with the target age group. Studies conducted exclusively among adolescents or adults were excluded.

**Exposure:** The primary exposure of interest was routine administration of two doses of measles-containing vaccine (MCV1 and MCV2) delivered through national Expanded Programme on Immunization schedules or equivalent routine childhood vaccination services. Studies involving single-dose vaccination, early or high-risk schedules, supplementary immunization activities, mixed routine/campaign exposures, revaccination design vaccine schedule comparisons, or special populations were retained when they reported relevant measles serological outcomes in the target population or a closely related age group. These studies were synthesized narratively and used to contextualize determinants, methodological variation, and evidence gaps.

**Outcomes:** The primary outcome was serological protection against measles, defined as the proportion of children with measles-specific antibody levels meeting the protective threshold defined by the individual study. Because laboratory assays, calibration standards, and protective thresholds varied across studies, serological protection estimates were interpreted according to the measurement method and threshold reported by each study rather than treated as directly interchangeable measures. Accepted laboratory measures included measles-specific IgG assays: enzyme-linked immunosorbent assay (ELISA), enzyme immunoassay (EIA), plaque reduction neutralization tests (PRNT), haemagglutination inhibition assay, particle agglutination assay, multiplex bead assay, or equivalent validated serological methods. Secondary outcomes included seropositivity, seroconversion, seronegativity, geometric mean concentration (GMC), geometric mean titer (GMT), antibody persistence, and reported determinants of serological protection or susceptibility. Determinants were grouped as biological, programmatic, temporal, and sociodemographic factors, where reported. Studies assessing only acute measles infection markers, such as IgM-only without vaccine-related serological protection data, were excluded.

**Study designs:** Eligible study designs included cross-sectional serosurveys, prevalence studies, prospective and retrospective cohort studies, randomized trials, comparative observational studies, quasi-experimental or pre/post vaccination studies, and programmatic immunization evaluations reporting laboratory-confirmed measles antibody outcomes. Case reports, very small case series, outbreak investigations without serological outcome data, modeling studies, qualitative studies, editorials, commentaries, narrative reviews, and conference abstracts without sufficient data were excluded.

**Setting:** Only studies conducted in LMICs were eligible. Studies from both community and health-facility settings were considered, including urban and rural populations. High-income country studies were excluded.

**Information sources and search strategy:** The literature search was conducted across six electronic databases: PubMed, MEDLINE, Scopus, Cochrane Library, CINAHL (EBSCO), and SciSpace (Milpitas CA, USA). The final search was conducted on 21 January 2026. Studies published in English between 2000 and 2026 were considered for inclusion. The search strategy combined controlled vocabulary and free-text terms related to measles, vaccination, immunity, serology, and LMIC settings. Complete database-specific search strings are provided in Table S2.

**Study selection:** All records identified through the search were imported into Rayyan.ai (Rayyan Systems, Inc., Cambridge, MA, USA), and duplicates were removed before screening. Titles and abstracts were screened against the predefined eligibility criteria, followed by full-text review of potentially eligible studies. Title/abstract screening and full-text eligibility assessment were undertaken independently by two reviewers. Disagreements were resolved through discussion and consensus between them. Where consensus could not be reached, a third reviewer was involved. The reasons for excluding studies at the full-text screening stage were recorded.

**Data extraction:** Data were extracted using a standardized data extraction form developed for this review. Extracted data were checked, harmonized, and organized for descriptive and narrative synthesis. The extraction form captured the following domains: study identification, country, WHO region, study design, setting, population characteristics, sample size, vaccination context, vaccination schedule, age at vaccination, age at serological assessment, time since vaccination, vaccination verification method, laboratory assay, seroprotection threshold, seroprotection or seropositivity estimates, seroconversion, seronegativity, GMC or GMT where reported, determinants of seroprotection or susceptibility, risk-of-bias information, and certainty-of-evidence judgments. Where studies reported multiple time points, vaccination groups, or population subgroups, data were extracted separately to preserve study-specific context. When denominators, thresholds, or timing of serological assessment were unclear, this was recorded as not reported or unclear rather than inferred.

**Decision framework for synthesis:** A structured decision framework was used to organize the evidence. First, studies were assessed for eligibility for inclusion in the systematic review based on the predefined population, exposure, setting, design, and outcome criteria. Second, included studies were categorized according to vaccination context, including routine two-dose vaccination, routine single-dose vaccination, early or high-risk schedules, mixed routine/campaign exposure, supplementary immunization activities, revaccination or schedule comparison studies, and special populations. Third, studies were grouped according to methodological features, including laboratory assay, seroprotection threshold, timing of serological assessment, vaccination verification, and risk of bias. This framework allowed the review to remain consistent with the registered protocol while avoiding inappropriate quantitative pooling of clinically and methodologically heterogeneous studies.

**Risk of bias assessment:** Risk of bias (RoB) was assessed using the Joanna Briggs Institute (JBI) critical appraisal tools, with the checklist selected according to study design [32,33,34,35]. Cohort studies were assessed using the JBI checklist for cohort studies, randomized trials using the JBI checklist for randomized controlled trials, cross-sectional analytical studies using the JBI analytical cross-sectional checklist, prevalence studies using the JBI prevalence checklist, and quasi-experimental or pre/post studies using the JBI quasi-experimental checklist. Studies were categorized as low, moderate, or high risk of bias based on selection bias, representativeness, vaccination exposure ascertainment, outcome measurement, confounding, follow-up completeness, and reporting clarity. Study-level risk-of-bias assessment, including overall judgement and domain-specific scores according to study design, is provided in Table S3.

**Certainty of evidence:** The certainty of evidence was assessed using the GRADE approach adapted for narrative synthesis [36]. Evidence was evaluated across the domains of risk of bias, inconsistency, indirectness, imprecision, and publication bias. Because most included studies were observational, descriptive, or heterogeneous in design and exposure definition, certainty judgments emphasized the coherence, consistency, directness, and methodological quality of the body of evidence rather than pooled effect estimates.

**Data synthesis:** Studies were grouped by vaccination context, including routine two-dose, single-dose vaccination, early vaccination schedules, supplementary immunization or campaign contexts, and special populations, including human immunodeficiency virus (HIV)-infected or HIV-exposed children. Findings were summarized descriptively using study-level seroprotection estimates, seroconversion where reported, antibody persistence, geometric mean concentration or titer where available, and reported determinants. Because seroprotection thresholds and assays varied across studies, estimates were interpreted within their original methodological context rather than treated as directly comparable effect estimates.

## 3. Results

### 3.1. Search Yield

**Figure 1:**
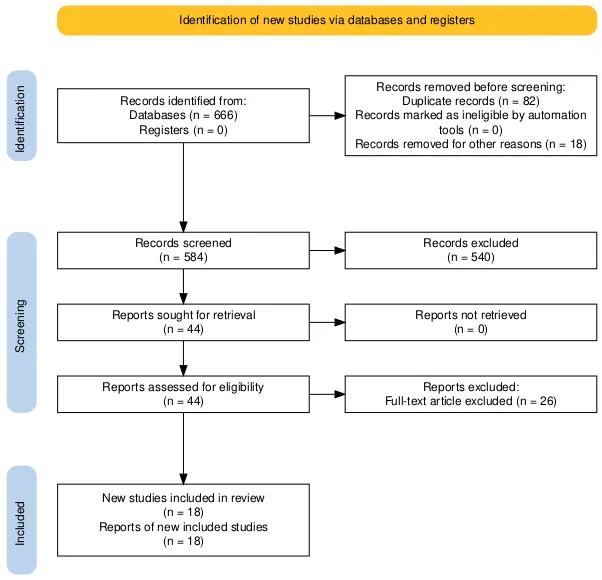
PRISMA 2020 flow diagram of study selection.

The literature search identified 666 records across the selected databases. After removal of duplicate records, 584 unique records remained for title and abstract screening. Subsequently, 540 records were excluded, and 44 full-text articles were retrieved and assessed for eligibility. Of these, 26 articles were excluded (for reasons including ineligible population, vaccination exposure, outcome measures, study design, or insufficient data) after full-text review, and 18 studies were finally included in the systematic review (Figure 1) (Table S4).

### 3.2. Descriptive Characteristics of Included Studies

The 18 included studies were published between 2001 and 2025 and were conducted across 14 LMICs representing 4 WHO regions. Most studies were from the African Region (*n* = 11; 61.1%), while fewer were from the Eastern Mediterranean (*n* = 5; 27.8%), South-East Asia (*n* = 1; 5.6%), and the Americas (*n* = 1; 5.6%) (Table 1).

The evidence was methodologically diverse. Study designs included prospective cohort studies, cross-sectional serosurveys, randomized vaccine schedule trials, comparative observational studies, and programmatic evaluations. Study populations included healthy children receiving routine immunization, children participating in supplementary immunization activities (SIAs), and biologically vulnerable groups, including HIV-infected and HIV-exposed children. Vaccination contexts also differed considerably across studies. Eight studies evaluated routine two-dose measles-containing vaccine schedules, four assessed routine single-dose vaccination, while the remaining studies examined early vaccination schedules, supplementary immunization activities, campaign-based vaccination, or comparative vaccine schedule strategies (Table 1).

### 3.3. Reported Serological Protection Across Studies

Across the 18 included studies, reported serological protection following measles-containing vaccination ranged from 30.6% to 100% (Table 2). The highest estimates were generally reported in studies assessing short-term responses after completion of a two-dose schedule, whereas lower estimates were observed in some single-dose, long-term follow-up, rural, and biologically vulnerable populations.

Studies that specifically evaluated routine two-dose vaccination schedules generally reported high seroprotection after the second dose. For instance, Saffar et al. [52], Hussain et al. [48], Martins et al. [42,43], and Verma et al. [53], reported seroprotection ranging from approximately 87–100% after completion of a second dose (Table 2). However, the magnitude of protection varied across settings, and short-term immunogenicity studies should not be interpreted as direct evidence of durable protection without longer follow-up.

Single-dose vaccination and long-term follow-up after a single dose were reported to have lower protection in the included studies, with evidence of waning immunity over time. Cross-sectional serosurveys also demonstrated marked geographic variation, with high seroprotection reported in Mexico, South Africa, Kenya, and Zambia, but considerably lower levels in Ethiopia and rural Pakistan. In the latter two countries, serological evidence of protection remained limited despite vaccination coverage improvements. These findings suggest that reported vaccination alone may be insufficient to infer population immunity in some LMIC settings.

Early two-dose vaccination schedules administered at 4.5–9 or 6–9 months produced high seroprotection among HIV-uninfected children (>90%), although antibody persistence was reduced compared with later vaccination schedules. In contrast, HIV-infected children consistently exhibited poorer and less durable immune responses, with seroprotection declining to 41% in one longitudinal study. Serological outcomes were assessed using various assays, including ELISA, PRNT, hemagglutination inhibition, particle agglutination, and immunofluorescence, with differing seroprotection thresholds. Despite this methodological heterogeneity, the evidence supports the superior immunogenicity of two-dose measles vaccination while emphasizing the influence of HIV infection, nutritional status, vaccination timing, and immunization programme performance on serological protection (Table 2).

### 3.4. Methodological Heterogeneity Across Studies

Methodological characteristics varied substantially across the included studies. Major sources of heterogeneity included study design, vaccination schedules, vaccination verification methods, participant selection, laboratory assays, seroprotection thresholds, timing of antibody assessment, and population. Laboratory assessment methods differed considerably, with ELISA/EIA being the most commonly used assay, while PRNT, hemagglutination inhibition, particle agglutination, multiplex bead assays, and filter-paper-based methods were also employed (Table S5). Because these assays measure different antibody characteristics and use different calibration systems, estimates could not be treated as directly comparable.

Across the included studies, reported seroprotection thresholds differed by assay type, ranging from ≥120 mIU/mL for PRNT-based assessments to 300–335 mIU/mL for some ELISA-based assessments; others used assay-specific cut-offs or non-standardized thresholds/definitions (Table S5). Timing of serological assessment ranged from short-term post-vaccination immunogenicity (4–8 weeks after vaccination) to long-term persistence studies extending up to 5 years following vaccination. Several cross-sectional studies lacked precise information on assessment time since vaccination. Additional heterogeneity arose from differences in study populations (e.g., HIV-infected children, campaign participants, or nationally representative samples), vaccination schedules, and programme settings (Table S5). These methodological differences limited direct comparison of seroprotection estimates and precluded quantitative synthesis, supporting the use of a structured narrative synthesis.

Within broadly comparable vaccination contexts, the included studies generally showed higher serological protection following completion of two measles-containing vaccine doses than after a single dose. However, the magnitude of this difference varied according to the timing of assessment, population characteristics, laboratory assay and seroprotection threshold, and therefore should not be interpreted as a pooled or directly comparable effect estimate.

### 3.5. Determinants of Measles Serological Protection

Determinants of measles serological protection varied across the studies and were broadly grouped into biological, programmatic, temporal, and sociodemographic factors (Table S6). Biological factors were most evident in studies involving HIV-infected and exposed children, where HIV infection was the most consistent determinant of reduced antibody response and poorer persistence. Poor nutritional status, including undernutrition and stunting, was associated with lower measles antibody concentrations in some studies, but the association was not assessed consistently across the evidence base.

Programmatic factors were more frequently evaluated than sociodemographic factors. Across studies, completion of a two-dose measles-containing vaccine schedule was consistently associated with higher and more durable serological protection than single-dose vaccination, and several studies reported improved antibody levels following revaccination with campaign doses. On the other hand, incomplete vaccination, delayed vaccination, uncertain vaccination documentation, and weak local programme performance were associated with lower measured protection in selected studies.

Moreover, short-term studies captured early immunogenicity, whereas longitudinal studies showed that antibody concentrations may decline over time, particularly among HIV-infected children or children with biological vulnerability. Sociodemographic determinants were assessed less consistently, although household crowding, unknown vaccination status, and rural residence were associated with increased susceptibility in selected population-based surveys (Table S6). Because most determinant analyses were observational, these findings should be interpreted as associations rather than causal effects.

### 3.6. Risk of Bias

Overall, the quality of the evidence was predominantly moderate, with 13 of the 18 studies judged to have a moderate risk of bias, four studies rated as high risk, and one study rated as low risk (Table 3 and Table S3). Prospective cohort studies and randomized vaccine schedule trials generally demonstrated stronger methodological quality than observational and pre–post studies, although incomplete reporting of allocation concealment, blinding, follow-up, and confounder adjustment frequently limited their ratings. RoB assessment was conducted using JBI critical appraisal checklists.

The principal methodological concerns across included studies were inadequate control of confounding, incomplete reporting of follow-up or attrition, limited vaccination verification, absence of control groups in pre–post designs, and restricted representativeness due to facility-based or special-population sampling. Cross-sectional studies were additionally limited by their inability to establish temporal relationships, while several older studies provided insufficient methodological detail for some JBI domains. Collectively, these limitations reduced confidence in direct comparisons between studies and further supported the use of a structured narrative synthesis rather than quantitative pooling of results.

### 3.7. Certainty of Evidence

The certainty of evidence ranged from low to very low across the evaluated outcomes (Table S7). Although most included studies consistently reported substantial serological protection following routine two-dose measles vaccination, confidence in the evidence was limited by predominantly observational study designs, methodological heterogeneity, inconsistent laboratory assays and seroprotection thresholds, variation in the timing of serological assessment, and risks of selection and measurement bias. Evidence regarding determinants of serological protection, including HIV infection, nutritional status, and assay-related variability, was further limited by inconsistency, indirectness, and imprecision. Consequently, the findings should be interpreted with caution and highlight the need for standardized serosurveillance methods and high-quality longitudinal studies to strengthen the evidence base in LMICs.

### 3.8. Evidence Gap

An evidence gap regarding serological protection following routine two-dose measles vaccination among children aged 1–5 years in LMICs was evident across the included studies. Few studies provided nationally representative estimates in LMICs, and most evidence came from sub-Saharan Africa (Table S8). Longitudinal data on antibody persistence after routine two-dose vaccination were limited. Vaccination verification was inconsistently reported, and few studies simultaneously assessed biological vulnerability, programme quality, nutritional status, and socioeconomic determinants. Notably, two South Asian countries (Pakistan nd Bangladesh) are now experiencing measles outbreaks, which were represented by only one included serosurvey [53], highlighting a geographic evidence gap [13,14,15,16]. Standardized assays, harmonized thresholds, and comparable reporting frameworks are needed to make future serosurveillance more useful for programme decision-making.

## 4. Discussion

This systematic review synthesized evidence on serological protection following measles-containing vaccination among children aged 1–5 years in LMICs. The available evidence indicates that routine two-dose measles vaccination generally induces substantial serological protection in young children. Reported levels of serological protection nonetheless varied considerably across studies, and this variation was driven primarily by differences in study populations, vaccination schedules, laboratory methods, seroprotection thresholds, timing of antibody assessment, and epidemiological contexts rather than vaccine performance alone.

A central finding of this review was that completion of routine two-dose measles vaccination was consistently associated with high levels of serological protection when administered according to recommended schedules. This finding is biologically plausible and consistent with the underlying purpose of the second dose of measles-containing vaccine, which provides an additional opportunity for seroconversion among children who fail to respond adequately to the first dose [1,4,6]. Several included studies reported high seroprotection after completion of the second dose, reinforcing the current recommendation of the WHO for routine two-dose Measles vaccination [2,25]. However, some studies reported lower serological protection despite a reported record of vaccination, indicating that a documented vaccination record or administrative coverage alone may not accurately reflect population immunity. Previous studies have emphasized that coverage estimates are vulnerable to inaccuracies in population denominators, reporting systems, and vaccination documentation and therefore cannot directly measure vaccine-induced immune protection [20,21]. Consistent with these observations, several included studies identified discrepancies between reported vaccination status and measured serological protection, suggesting that serosurveillance can provide valuable complementary information for identifying immunity gaps and evaluating immunization programme performance. Serological assessment therefore offers a complementary tool for identifying immunity gaps that may not be apparent from routine coverage data alone, particularly in settings approaching measles elimination.

The discrepancy between reported vaccination coverage and actual serological protection is increasingly evident in South Asia. In Pakistan, the 2026 measles outbreak occurred despite a long-standing two-dose routine immunization schedule, complemented by repeated nationwide supplementary immunization activities that reportedly vaccinated more than 232.5 million children between 2005 and 2018 [13]. Similarly, the outbreak in Bangladesh occurred despite historically high MCV1 coverage. However, MCV2 coverage declined from 89.0% to 80.7% over five years, leaving an estimated 20 million children without adequate second-dose protection—a gap that was not detected through routine surveillance before the outbreak [15,16]. Although biological vaccine failure may have contributed to susceptibility at the individual level, the scale of these outbreaks is more consistent with the progressive accumulation of unrecognized population-level immunity gaps, reflecting the determinants and methodological limitations identified in this review. These findings support the integration of standardized serological surveillance into routine immunization programmes. Used alongside administrative coverage data, serological monitoring could provide earlier warning of population susceptibility and should be prioritized in LMICs approaching, but not yet sustaining, measles elimination. The review also identified several biological and programmatic determinants that may influence vaccine-derived immunity. Several studies involving HIV-infected or HIV-exposed children reported lower serological protection or more rapid decline in antibody levels, consistent with previous evidence showing impaired humoral immune responses among immunocompromised populations [55]. Similarly, undernutrition and stunting were associated with lower measles antibody concentrations in some studies, supporting broader evidence that malnutrition can weaken immune function and vaccine responsiveness [56,57]. However, the magnitude and consistency of this association varied across settings, partly because nutritional status was not assessed uniformly across studies. These findings emphasize that biological vulnerability should be considered when interpreting serological protection in LMIC populations, particularly where HIV infection and childhood malnutrition remain prevalent.

Timeliness of vaccination was another important determinant of serological response. Included studies reported lower serological protection among children vaccinated at younger ages, consistent with existing evidence that residual maternal antibodies can neutralize vaccine virus replication and reduce seroconversion when vaccination is administered before maternal antibodies have sufficiently declined [18,19]. Conversely, delaying vaccination may increase the risk of measles infection by extending the period during which infants remain susceptible. These findings reinforce the rationale for WHO-recommended vaccination schedules, which seek to balance early protection with optimal vaccine immunogenicity according to local epidemiology [6,17]. They also highlight the importance of timely completion of both vaccine doses, as incomplete or delayed vaccination may allow susceptibility to persist in young children.

Programmatic factors were found to further influence reported immunity in the included studies. Delayed vaccination, incomplete vaccination schedules, inadequate follow-up, and missed opportunities for immunization were repeatedly associated with lower serological protection, reflecting broader challenges within routine immunization programmes [8,18]. In several settings, supplementary immunization activities (SIAs) contributed to improved immunity by reaching previously unvaccinated children and reducing susceptibility within high-risk populations. Nevertheless, SIAs should complement rather than replace strong routine immunization systems, as sustainable measles elimination depends on consistently achieving high coverage with timely administration of both routine vaccine doses [4,6].

An important finding of this review was the substantial methodological heterogeneity across the included studies, which limited direct comparison of reported serological protection. Although all studies evaluated measles vaccine-induced immunity, they differed considerably in study design, participant selection, vaccination schedules, laboratory assays, seroprotection thresholds, timing of serological assessment, and vaccination verification methods. Consequently, variation in reported serological protection should be interpreted cautiously, as it may reflect methodological differences rather than true variation in vaccine performance [7,20,21]. Most studies measured measles-specific antibodies using ELISA or EIA, whereas others employed PRNT, haemagglutination inhibition assays, particle agglutination assays, or multiplex bead-based assays. While ELISA-based methods are practical for large-scale serosurveillance, they quantify binding antibodies rather than functional neutralizing antibodies, whereas PRNT is considered the laboratory reference standard for assessing protective immunity [6,20]. Differences in assay performance, calibration, sensitivity, and specificity therefore likely contributed to the variability in reported serological protection across studies [27,29,58,59].

In addition, the commonly used threshold for measles antibodies is itself not absolute. Although a neutralizing antibody concentration of approximately 120 mIU/mL is commonly regarded as a correlate of protection, several studies applied higher thresholds or assay-specific cut-offs that were not directly comparable [21,29]. These inconsistencies complicate comparisons between studies and highlight the need for harmonized laboratory standards and reporting practices to improve the interpretation of serological data. Previous reviews have also highlighted that differences in laboratory methods and reporting units complicate comparisons across studies [60]. Similarly, the timing of antibody measurement also differed substantially across studies. Some studies assessed serological responses within weeks of vaccination, reflecting short-term immunogenicity, whereas others measured antibody persistence several years after completion of the vaccination schedule. Because antibody concentrations naturally change over time, differences in follow-up duration may influence estimates of serological protection independently of vaccine effectiveness. Similarly, variation in vaccination verification methods, including reliance on immunization cards, clinic records, registries, or parental recall, introduced additional uncertainty regarding vaccination status and may have contributed to exposure misclassification in some observational studies.

The findings of this review have important implications for measles control and elimination efforts in LMICs. They reinforce the importance of achieving timely and equitable coverage with routine measles-containing vaccine doses. They also indicate that vaccination coverage should be interpreted alongside serological and epidemiological data, particularly in settings with recurrent outbreaks despite apparently high coverage. Moreover, they highlight the need to focus on vulnerable populations, including under-immunized children, children affected by malnutrition, children living with or exposed to HIV, and children in conflict-affected, displaced, or geographically remote communities. In such settings, serosurveillance may help identify susceptible age groups and guide targeted immunization strategies before outbreaks occur [6,20,23].

These findings are relevant to the goals of the Immunization Agenda 2030 and the WHO Measles and Rubella Strategic Framework 2021–2030, both of which emphasize equitable immunization coverage, data-driven programme improvement, and stronger surveillance systems as prerequisites for achieving and sustaining measles elimination [2,25]. Strengthening routine two-dose vaccination while improving the quality and standardization of serological monitoring will be essential for identifying immunity gaps, preventing outbreaks, and supporting evidence-based immunization policies in LMICs.

Several limitations should be considered. The included studies were highly heterogeneous with respect to study design, participant characteristics, vaccination schedules, laboratory assays, seroprotection thresholds, vaccination verification, and timing of serological assessment. Consequently, quantitative synthesis was considered inappropriate, and findings were synthesized narratively. The evidence was also geographically uneven, with most studies originating from sub-Saharan Africa, limiting generalizability to all LMICs. Variation in laboratory methods and inconsistent reporting of vaccination history and potential confounders further reduced comparability across studies. In addition, much of the broader evidence base was observational, descriptive, or based on mixed exposure histories, which limits causal interpretation. Due to the predominance of observational study designs and considerable methodological heterogeneity, these findings cannot establish causality and may lack generalizability beyond the studied populations. Determinant analyses were also largely exploratory and often unadjusted, meaning that observed associations may be affected by confounding. Variability in assay methods, seroprotection thresholds, and timing of sample collection further reduced comparability across studies, a challenge widely recognized in measles sero-surveillance research [20,21,61,62]. Despite these limitations, the consistency of findings across diverse settings supports the overall conclusion that routine two-dose measles vaccination provides substantial serological protection while highlighting important methodological and evidence gaps requiring further investigation. Overall, the findings suggest that routine two-dose measles vaccination may achieve high serological protection in some LMIC settings, but the directly relevant evidence base remains sparse, heterogeneous, and too limited to support firm conclusions across LMICs.

## 5. Conclusions

Routine two-dose measles-containing vaccination generally provides substantial serological protection among children aged 1–5 years in LMICs, supporting its continued role as the cornerstone of measles control and elimination programmes. However, variation in reported serological protection was observed across studies, reflecting a combination of biological determinants (age at vaccination, HIV status, nutritional status), programmatic factors (delayed or incomplete vaccination, health-system performance), and, most importantly, methodological heterogeneity in laboratory assays, seroprotection thresholds, and timing of assessment. This heterogeneity, rather than vaccine performance alone, accounts for much of the observed variability and currently limits the overall certainty of the evidence.

These findings highlight the need for harmonized serosurveillance methodologies, standardized laboratory reporting, and well-designed longitudinal studies to improve understanding of vaccine-derived immunity following routine measles vaccination. Simultaneously, integrating standardized serological surveillance into routine immunization monitoring along with efforts to strengthen timely measles two-dose vaccination delivery will be essential for identifying immunity gaps, guiding evidence-informed vaccination policies, and supporting progress toward global measles elimination.

## Figures and Tables

**Table 1:** Descriptive characteristics of included studies.

Domain	Category	Count (*n*)	Percentage (%)	Supporting Studies
WHO Region	African Region (AFRO)	11	61.1	(Campbell et al. 2021; Eskenazi et al. 2025; Fowlkes et al. 2011; Helfand et al. 2008; Kanga 2021; Martins et al. 2013, 2014; Prosperi et al. 2025; Sudfeld et al. 2013; Takechi et al. 2001; Tejiokem et al. 2025) [37,38,39,40,41,42,43,44,45,46,47].
	Eastern Mediterranean Region (EMRO)	5	27.8	(Hussain et al. 2013, 2024; Ibrahim et al. 2006; Karwi et al. 2020; Saffar et al. 2011) [48,49,50,51,52].
	South-East Asia Region (SEARO)	1	5.6	(Verma et al. 2023) [53].
	Region of the Americas (AMRO)	1	5.6	(Díaz-Ortega et al. 2020) [54].
Study Design	Cross-sectional	7	38.9	(Campbell et al. 2021; Kanga 2021; Prosperi et al. 2025; Takechi et al. 2001; Hussain et al. 2024; Karwi et al. 2020; Díaz-Ortega et al. 2020) [37,41,44,46,49,51,54].
	Prospective cohort/Longitudinal	5	27.8	(Eskenazi et al. 2025; Fowlkes et al. 2011; Tejiokem et al. 2025; Ibrahim et al. 2006;Verma et al. 2023) [38,39,47,50,53]
	Randomized trial/schedule trial	4	22.2	(Helfand et al. 2008; Martins et al. 2013, 2014; Sudfeld et al. 2013) [40,42,43,45]
	Comparative observational	2	11.1	(Hussain et al. 2013; Saffar et al. 2011) [48,52]
Setting	Community/population-based	9	50.0	(Campbell et al. 2021; Eskenazi et al. 2025; Martins et al. 2013, 2014; Prosperi et al. 2025; Takechi et al. 2001; Hussain et al. 2024; Ibrahim et al. 2006; Díaz-Ortega et al. 2020) [37,38,42,43,44,46,49,50,54]
	Health facility/clinic/hospital	9	50.0	(Fowlkes et al. 2011; Helfand et al. 2008; Kanga 2021; Sudfeld et al. 2013; Tejiokem et al. 2025; Hussain et al. 2013; Karwi et al. 2020; Saffar et al. 2011; Verma et al. 2023) [39,40,41,45,47,48,51,52,53]
Vaccination context	Routine	9	50.0	(Eskenazi et al. 2025; Martins et al. 2013; Hussain et al. 2013, 2024; Ibrahim et al. 2006; Karwi et al. 2020; Saffar et al. 2011; Verma et al. 2023; Díaz-Ortega et al. 2020) [38,42,48,49,50,51,52,53,54]
	Early schedule	2	11.1	(Helfand et al. 2008; Martins et al. 2013) [40,42]
	Special populations	3	16.7	(Fowlkes et al. 2011; Sudfeld et al. 2013; Tejiokem et al. 2025) [39,45,47]
	Campaign	2	11.1	(Prosperi et al. 2025; Takechi et al. 2001) [44,46]
	Mixed routine/campaign	1	5.6	(Campbell et al. 2021) [37]
	Early + routine	1	5.6	(Tejiokem et al. 2025) [47]
Population	General healthy children	10	55.6	(Campbell et al. 2021; Eskenazi et al. 2025; Kanga 2021; Martins et al. 2013, 2014; Takechi et al. 2001; Hussain et al. 2013; Karwi et al. 2020; Saffar et al. 2011; Verma et al. 2023) [37,38,41,42,43,46,48,51,52,53]
	HIV-related	4	22.2	(Fowlkes et al. 2011; Helfand et al. 2008; Sudfeld et al. 2013; Tejiokem et al. 2025) [39,40,45,47]
	General population	1	5.6	(Díaz-Ortega et al. 2020) [54]
	Rural community children	2	11.1	(Hussain et al. 2024; Ibrahim et al. 2006) [49,50]
	Mixed/special schedule	1	5.6	(Prosperi et al. 2025) [44]

**Table 2:** Reported serological outcome across included studies.

Study	Country	Vaccination Context	Vaccination Schedule	Study-Specific Serological Outcome (%)	Key Finding
Takechi et al., 2001 [46]	Malawi	Campaign	Mass measles campaign	100 (seroconversion)	Campaign induced complete seroconversion among initially seronegative children.
Ibrahim et al., 2006 [50]	Sudan	Routine	MCV1 at 9 months	45–85	Antibody levels increased with age following vaccination.
Helfand et al., 2008 [40]	Malawi	Early/HIV	6 + 9 months	92–94 (HIV−); 64 (HIV+)	HIV infection substantially reduced vaccine response.
Saffar et al., 2011 [52]	Iran	Routine	MMR2 at 18 months or 4–6 years	94–98.2	Second dose markedly increased immunity.
Fowlkes et al., 2011 [39]	Malawi	Early/HIV	6 + 9 months	84–87 (HIV−); 41 (HIV+)	Protection persisted in HIV-uninfected children but declined markedly in HIV-infected children.
Hussain et al., 2013 [48]	Pakistan	Routine	One vs. two doses	73 (one dose); 87 (two doses); 63 (5-year follow-up)	Two-dose schedule provided superior and more durable protection.
Martins et al., 2013 [42]	Guinea-Bissau	Routine/Early	9 months ±18 months	99	Booster increased antibody titres despite already high protection.
Martins et al., 2014 [43]	Guinea-Bissau	Early	4.5 + 9 months vs. 9 months	97–99	Two-dose schedules achieved near-complete protection by 24 months.
Díaz-Ortega et al., 2020 [54]	Mexico	Routine	National MMR schedule	99.4	National seroprotection exceeded the elimination threshold in most age groups.
Karwi et al., 2020 [51]	Iraq	Routine	Second MCV	92.4	Second dose substantially improved immunity.
Campbell et al., 2021 [37]	Ethiopia	Routine	MCV1 ± campaign	40–54	Persistent immunity gaps despite programme improvements.
Kanga, 2021 [41]	Kenya	Routine	Routine MCV	87.4	Immunity below herd immunity target; vaccination documentation strongly associated with protection.
Verma et al., 2023 [53]	India	Routine	MR1 + MR2	88.7 after MR1; 100 after MR2	Complete seroprotection achieved after second dose.
Hussain et al., 2024 [49]	Pakistan	Routine	Routine vaccination	30.6	Very low detectable immunity despite reported vaccination.
Eskenazi et al., 2025 [38]	South Africa	Routine	MCV1 + MCV2	90.4–91.9	Most children remained protected; undernutrition reduced antibody levels.
Prosperi et al., 2025 [44]	Zambia	Campaign	SIA + routine MR	86	Campaign improved coverage but immunity remained below the 95% elimination target.
Tejiokem et al., 2025 [47]	Cameroon	Early/HIV	6 + 9 months + MMR	87–100	High initial protection with gradual waning over time.
Sudfeld et al., 2013 [45]	Tanzania	HIV-exposed	Routine MCV1	68.7	Moderate protection influenced by nutritional status and maternal HIV severity.

Note: Serological protection was defined according to the study-specific laboratory assay and protective threshold. Detailed information on laboratory assays, seroprotection thresholds/definitions, and timing of antibody assessment is provided in Table S5.

**Table 3:** Risk of bias assessment of included studies.

Studies	JBI Checklist Used	Overall RoB	Key Methodological Concerns
Díaz-Ortega et al., 2020 [54]	Analytical cross-sectional	Low	Minor limitation inherent to cross-sectional design; otherwise methodologically strong
Tejiokem et al., 2025 [47]	Cohort	Moderate	Clinic-based cohort; no confounder adjustment; follow-up completeness not fully analysed
Eskenazi et al., 2025 [38]	Cohort	Moderate	Cohort restricted to fully vaccinated children; vaccination verification incompletely described in article text
Campbell et al., 2021 [37]	Prevalence	Moderate	Good cluster sampling, but descriptive analysis only and mixed campaign/routine exposure
Ibrahim et al., 2006 [50]	Cohort	Moderate	Vaccination history mainly by parental report; no confounder handling
Sudfeld et al., 2013 [45]	RCT	Moderate	Good randomization and masking, but subset outcome data and some attrition
Martins et al., 2013 [42]	RCT	Moderate	Allocation via envelope described, but concealment/blinding incompletely reported; attrition present
Prosperi et al., 2025 [44]	Prevalence	Moderate	Campaign-participant sample rather than full population frame; limited adjustment
Saffar et al., 2011 [52]	Quasi-experimental	Moderate	No contemporary control group; blinding not reported
Hussain et al., 2013 [48]	Analytical cross-sectional	Moderate	Non-randomized grouped comparison; serial/household recruitment; no confounder control
Verma et al., 2023 [53]	Cohort	Moderate	Single-center consecutive sample; no control group; duplicate testing not performed
Martins et al., 2014 [43]	RCT	Moderate	Block randomization well described, but blinding and attrition handling incompletely detailed
Helfand et al., 2008 [40]	RCT	Moderate	Block randomization reported, but concealment/blinding incompletely described; follow-up losses present
Fowlkes et al., 2011 [39]	Cohort	Moderate	Follow-up study from prior trial; no confounder adjustment; incomplete-follow-up strategy limited
Takechi et al., 2001 [46]	Quasi-experimental	High	No control group; campaign-attendee sample; assay reporting limited
Kanga, 2021 [41]	Analytical cross-sectional	High	Facility-based consecutive sampling; no confounder assessment; limited external validity
Karwi et al., 2020 [51]	Quasi-experimental	High	Reporting incomplete; no control group; limited statistical detail
Hussain et al., 2024 [49]	Analytical cross-sectional	High	Sampling method insufficiently described; vaccination verified by parental record; no multivariable control

Note: Color-coded text indicates the overall risk-of-bias (RoB) judgment: green = low risk of bias, orange = moderate risk of bias, and red = high risk of bias.

## Data Availability

The data presented in this study are available on request from the corresponding author.

## Supplementary Materials

The following supporting information can be downloaded at: https://www.mdpi.com/article/10.3390/vaccines14090760/s1, Table S1. PRISMA 2020 Checklist; Table S2. Search Strategy; Table S3. Study-level risk-of-bias assessment, including overall judgement and domain-specific scores according to study design; Table S4. Full-text articles assessed for eligibility (*n* = 44), with final inclusion decision and reason; Table S5. Summary of laboratory methods, seroprotection thresholds, and methodological heterogeneity across included studies; Table S6. Study-level determinants of measles serological protection across included studies; Table S7. Certainty of evidence for serological protection outcomes following routine two-dose measles vaccination in children 1–5 years in LMICs—Using GRADE; Table S8. Evidence Gaps identified across the included studies.

## Abbreviations

The following abbreviations are used in this manuscript:

AFRO	WHO African Region
AMRO	WHO Region of the Americas
CINAHL	Cumulative Index to Nursing and Allied Health Literature
EIA	Enzyme immunoassay
ELISA	Enzyme-linked immunosorbent assay
EMRO	WHO Eastern Mediterranean Region
EPI	Expanded Programme on Immunization
GMC	Geometric mean concentration
GMT	Geometric mean titre
GRADE	Grading of Recommendations Assessment, Development and Evaluation
HIV	Human immunodeficiency virus
IgG	Immunoglobulin G
IgM	Immunoglobulin M
IU/mL	International units per millilitre
JBI	Joanna Briggs Institute
LMICs	Low- and middle-income countries
MBA	Multiplex bead assay
MCV	Measles-containing vaccine
MCV1	First dose of measles-containing vaccine
MCV2	Second dose of measles-containing vaccine
MEDLINE	Medical Literature Analysis and Retrieval System Online
mIU/mL	Milli-international units per millilitre
MMR	Measles–mumps–rubella vaccine
MMR2	Second dose of measles–mumps–rubella vaccine
MR	Measles–rubella vaccine
MR1	First dose of measles–rubella vaccine
MR2	Second dose of measles–rubella vaccine
NR	Not reported
PICO	Population, intervention or exposure, comparator, and outcome
PRISMA	Preferred Reporting Items for Systematic Reviews and Meta-Analyses
PRNT	Plaque reduction neutralization test
PROSPERO	International Prospective Register of Systematic Reviews
R_0_	Basic reproduction number
RCT	Randomized controlled trial
RoB	Risk of bias
SEARO	WHO South-East Asia Region
SIA	Supplementary immunization activity
SIAs	Supplementary immunization activities
WHO	World Health Organization
