# Supplementary material for: Serological Protection After Routine Measles-Containing Vaccination Among Children Aged 1–5 Years in Low- and Middle-Income Countries: A Systematic Review
Source: Vaccines (Basel). 2026 Aug 31;14(9):760. doi: 10.3390/vaccines14090760 (PMC13611754; doi:10.3390/vaccines14090760)
Supplement: Supplementary file 1 [file vaccines-14-00760-s001.zip › vaccines-4288402-supplementary.pdf]

Table S1. PRISMA 2020 Checklist

Section and Topic	Item #	Checklist item	Location where item is reported
**TITLE**			
Title	1	Identify the report as a systematic review.	Page 1
**ABSTRACT**			
Abstract	2	See the PRISMA 2020 for Abstracts checklist.	Page 1
**INTRODUCTION**			
Rationale	3	Describe the rationale for the review in the context of existing knowledge.	Page 2
Objectives	4	Provide an explicit statement of the objective(s) or question(s) the review addresses.	Page 4
**METHODS**			
Eligibility criteria	5	Specify the inclusion and exclusion criteria for the review and how studies were grouped for the syntheses.	Page 4
Information sources	6	Specify all databases, registers, websites, organisations, reference lists and other sources searched or consulted to identify studies. Specify the date when each source was last searched or consulted.	Page 5
Search strategy	7	Present the full search strategies for all databases, registers and websites, including any filters and limits used.	Page 5
Selection process	8	Specify the methods used to decide whether a study met the inclusion criteria of the review, including how many reviewers screened each record and each report retrieved, whether they worked independently, and if applicable, details of automation tools used in the process.	Pages 4 &5
Data collection process	9	Specify the methods used to collect data from reports, including how many reviewers collected data from each report, whether they worked independently, any processes for obtaining or confirming data from study investigators, and if applicable, details of automation tools used in the process.	Pages 4 &5
Data items	10a	List and define all outcomes for which data were sought. Specify whether all results that were compatible with each outcome domain in each study were sought (e.g. for all measures, time points, analyses), and if not, the methods used to decide which results to collect.	Pages 4 &5
	10b	List and define all other variables for which data were sought (e.g. participant and intervention characteristics, funding sources). Describe any assumptions made about any missing or unclear information.	Pages 5
Study risk of bias assessment	11	Specify the methods used to assess risk of bias in the included studies, including details of the tool(s) used, how many reviewers assessed each study and whether they worked independently, and if applicable, details of automation tools used in the process.	Page 5 &6
Effect measures	12	Specify for each outcome the effect measure(s) (e.g. risk ratio, mean difference) used in the synthesis or presentation of results.	Page 5 &6
Synthesis methods	13a	Describe the processes used to decide which studies were eligible for each synthesis (e.g. tabulating the study intervention characteristics and comparing against the planned groups for each synthesis (item #5)).	Pages 5 &6
	13b	Describe any methods required to prepare the data for presentation or synthesis, such as handling of missing summary statistics, or data conversions.	Pages 5 &6
	13c	Describe any methods used to tabulate or visually display results of individual studies and syntheses.	Pages 5 &6
	13d	Describe any methods used to synthesize results and provide a rationale for the choice(s). If meta-analysis was performed, describe the model(s), method(s) to identify the presence and extent of statistical heterogeneity, and software package(s) used.	Pages 5 &6
	13e	Describe any methods used to explore possible causes of heterogeneity among study results (e.g. subgroup analysis, meta-regression).	Pages 5 &6
	13f	Describe any sensitivity analyses conducted to assess robustness of the synthesized results.	Pages 5 &6
Reporting bias assessment	14	Describe any methods used to assess risk of bias due to missing results in a synthesis (arising from reporting biases).	Pages 5 &6
Certainty assessment	15	Describe any methods used to assess certainty (or confidence) in the body of evidence for an outcome.	Pages 5 &6
**RESULTS**			

Section and Topic	Item #	Checklist item	Location where item is reported
Study selection	16a	Describe the results of the search and selection process, from the number of records identified in the search to the number of studies included in the review, ideally using a flow diagram.	Pages 6&7
	16b	Cite studies that might appear to meet the inclusion criteria, but which were excluded, and explain why they were excluded.	Page 6
Study characteristics	17	Cite each included study and present its characteristics.	Pages 7 &8
Risk of bias in studies	18	Present assessments of risk of bias for each included study.	Pages 13 &14
Results of individual studies	19	For all outcomes, present, for each study: (a) summary statistics for each group (where appropriate) and (b) an effect estimate and its precision (e.g. confidence/credible interval), ideally using structured tables or plots.	Pages 7, 8&9
Results of syntheses	20a	For each synthesis, briefly summarise the characteristics and risk of bias among contributing studies.	Pages 13 &14
	20b	Present results of all statistical syntheses conducted. If meta-analysis was done, present for each the summary estimate and its precision (e.g. confidence/credible interval) and measures of statistical heterogeneity. If comparing groups, describe the direction of the effect.	NA
	20c	Present results of all investigations of possible causes of heterogeneity among study results.	Pages 12&13
	20d	Present results of all sensitivity analyses conducted to assess the robustness of the synthesized results.	NA
Reporting biases	21	Present assessments of risk of bias due to missing results (arising from reporting biases) for each synthesis assessed.	NA
Certainty of evidence	22	Present assessments of certainty (or confidence) in the body of evidence for each outcome assessed.	Pages 13 &14
**DISCUSSION**			
Discussion	23a	Provide a general interpretation of the results in the context of other evidence.	Page 15
	23b	Discuss any limitations of the evidence included in the review.	Page 18
	23c	Discuss any limitations of the review processes used.	Page 18
	23d	Discuss implications of the results for practice, policy, and future research.	Pages 18
**OTHER INFORMATION**			
Registration and protocol	24a	Provide registration information for the review, including register name and registration number, or state that the review was not registered.	Page 4
	24b	Indicate where the review protocol can be accessed, or state that a protocol was not prepared.	Page 4
	24c	Describe and explain any amendments to information provided at registration or in the protocol.	Page 4
Support	25	Describe sources of financial or non-financial support for the review, and the role of the funders or sponsors in the review.	Page 19
Competing interests	26	Declare any competing interests of review authors.	Page 19
Availability of data, code and other materials	27	Report which of the following are publicly available and where they can be found: template data collection forms; data extracted from included studies; data used for all analyses; analytic code; any other materials used in the review.	Page 19

From: Page MJ, McKenzie JE, Bossuyt PM, Boutron I, Hoffmann TC, Mulrow CD, et al. The PRISMA 2020 statement: an updated guideline for reporting systematic reviews. BMJ 2021;372:n71. doi: 10.1136/bmj.n71. This work is licensed under CC BY 4.0. To view a copy of this license, visit https://creativecommons.org/licenses/by/4.0/

Table S2. Search Strategy

PubMed Mesh Terms:<br>("Measles"[MeSH] OR "measles virus"[MeSH] OR measles[tiab] OR "measles virus"[tiab])<br>AND<br>("Measles Vaccines"[MeSH] OR "measles vaccine*"[tiab] OR vaccination[tiab] OR "measles-containing vaccine"[tiab] OR MCV[tiab] OR "measles vaccination")<br>AND<br>("Antibodies, Viral"[MeSH] OR "Serologic Tests"[MeSH] OR seroprotection[tiab] OR seroprevalence[tiab] OR IgG[tiab] OR antibody[tiab] OR seroconversion[tiab] OR "immune response"[tiab] OR immunity[tiab])<br>AND<br>("Child, Preschool"[MeSH] OR "Infant"[MeSH] OR "Child"[MeSH] OR child*[tiab] OR infant*[tiab] OR preschool[tiab] OR toddler*[tiab] OR "underfive"[tiab] OR "under 5"[tiab])<br>AND<br>("Developing Countries"[MeSH] OR "Low- and Middle-Income Countries"[tiab] OR LMIC[tiab] OR "Africa"[tiab] OR "Asia"[tiab] OR "Latin America"[tiab] OR "South Asia"[tiab])
Google Scholar:<br>All in title: "measles" "measles-containing vaccine" "two-dose" ("seroprotection" OR "seroprevalence" OR "IgG" OR "antibody" OR "seroconversion") ("child" OR "infant" OR "preschool") ("Afghanistan" OR "Bangladesh" OR "Benin" OR "Bhutan" OR "Burkina Faso" OR "Burundi" OR "Cambodia" OR "Cameroon" OR "Central African Republic" OR "Chad" OR "Comoros" OR "Congo" OR "Côte d'Ivoire" OR "Djibouti" OR "Democratic Republic of the Congo" OR "Eritrea" OR "Ethiopia" OR "Gambia" OR "Ghana" OR "Guinea" OR "Guinea-Bissau" OR "Haiti" OR "India" OR "Indonesia" OR "Kenya" OR "Kiribati" OR "Kyrgyzstan" OR "Lao People's Democratic Republic" OR "Lesotho" OR "Liberia" OR "Madagascar" OR "Malawi" OR "Mali" OR "Mauritania" OR "Mozambique" OR "Myanmar" OR "Nepal" OR "Niger" OR "Nigeria" OR "Pakistan" OR "Papua New Guinea" OR "Rwanda"
Scopus<br>(TITLE-ABS-KEY ((Measles OR "measles virus")) AND TITLE-ABS-KEY (("Measles Vaccine*" OR vaccination OR "measles-containing vaccine" OR MCV OR "measles vaccination")) AND TITLE-ABS-KEY (("Serologic Test*" OR seroprotection OR seroprevalence OR IgG OR antibod* OR seroconversion OR "immune response" OR immunity)) AND TITLE-ABS-KEY ((child* OR infant* OR preschool OR toddler* OR "under five" OR "under 5" OR underfive)) AND TITLE-ABS-KEY (("Developing Countries" OR "Low- and Middle-Income Countr*" OR LMIC OR Africa OR Asia OR "Latin America" OR "South Asia")))<br>531 results
CINAHL<br>((TI (measles OR "measles virus") OR AB (measles OR "measles virus")) AND<br>(TI ("measles vaccine*" OR vaccination OR "measles-containing vaccine" OR MCV OR "measles vaccination") OR AB ("measles vaccine*" OR vaccination OR "measles-containing vaccine" OR MCV OR "measles vaccination")) AND (TI ("serologic test*" OR seroprotection OR seroprevalence OR IgG OR antibod* OR seroconversion OR "immune response" OR immunity) OR AB ("serologic test*" OR seroprotection OR seroprevalence OR IgG OR antibod* OR seroconversion OR "immune response" OR immunity)) AND<br>(TI (child* OR infant* OR preschool* OR toddler* OR "under five" OR "under 5" OR underfive) OR AB (child* OR infant* OR preschool* OR toddler* OR "under five" OR "under 5" OR underfive)) AND<br>(TI ("developing countr*" OR "low- and middle-income countr*" OR LMIC* OR Africa OR Asia OR "Latin America" OR "South Asia") OR AB ("developing countr*" OR "low- and middle-income countr*" OR LMIC* OR Africa OR Asia OR "Latin America" OR "South Asia")))<br>19 results
Cochrane<br>Key words only:<br>(Measles OR "measles virus")<br>AND ("Measles Vaccine*" OR vaccination OR "measles-containing vaccine" OR MCV OR "measles vaccination")<br>AND ("Serologic Test*" OR seroprotection OR seroprevalence OR IgG OR antibod* OR seroconversion OR "immune response" OR immunity)<br>AND (child* OR infant* OR preschool OR toddler* OR "under five" OR "under 5" OR underfive)<br>AND ("Developing Countries" OR "Low- and Middle-Income Countr*" OR LMIC OR Africa OR Asia OR "Latin America" OR "South Asia")

Table S3. Study-level risk-of-bias assessment, including overall judgement and domain-specific scores according to study design

JBI Cohort Checklist Item-Level Risk of Bias													
Citation	Overall RoB	C1: Similar groups recruited from same population	C2: Exposures measured similarly to assign groups	C3: Exposure measured validly and reliably	C4: Confounding factors identified	C5: Strategies to deal with confounding stated	C6: Groups/participants free of outcome at baseline	C7: Outcomes measured validly and reliably	C8: Follow-up time sufficient	C9: Follow-up complete or losses described	C10: Strategies to address incomplete follow-up	C11: Appropriate statistical analysis	Key methodological concerns
Tejiokem et al., 2025	Moderate	Y	Y	Y	N	N	U	Y	Y	U	U	Y	Clinic-based cohort; no confounder adjustment; follow-up completeness not fully analysed
Eskenazi et al., 2025	Moderate	Y	Y	Y	Y	Y	U	Y	Y	Y	Y	Y	Cohort restricted to fully vaccinated children; vaccination verification incompletely described in article text
Ibrahim et al., 2006	Moderate	Y	Y	Y	N	N	N	Y	Y	Y	N	Y	Vaccination history mainly by parental report; no confounder handling
Verma et al., 2023	Moderate	Y	Y	Y	N	N	U	Y	Y	U	U	Y	Single-center consecutive sample; no control group; duplicate testing not performed
Fowlkes et al., 2011	Moderate	Y	Y	Y	N	N	U	Y	Y	Y	U	Y	Follow-up study from prior trial; no confounder adjustment; incomplete-follow-up strategy limited

JBI RCT Checklist Item-Level Risk of Bias															
Citation	Overall RoB	R1: True randomization used	R2: Allocation concealed	R3: Treatment groups similar at baseline	R4: Participants blind to treatment assignment	R5: Treatment deliverers blind	R6: Outcome assessors blind	R7: Groups treated identically other than intervention	R8: Follow-up complete; differences described/analysed	R9: Participants analysed in randomized groups	R10: Outcomes measured same way	R11: Outcomes measured reliably	R12: Appropriate statistical analysis	R13: Trial design appropriate; deviations accounted for	Key methodological concerns
Sudfeld et al., 2013	Moderate	Y	U	Y	Y	Y	U	Y	U	Y	Y	Y	Y	Y	Good randomization and masking, but subset outcome data and some attrition
Martins et al., 2013	Moderate	Y	U	Y	U	U	U	Y	U	Y	Y	Y	Y	Y	Allocation via envelope described, but concealment/blinding incompletely reported; attrition present
Martins et al., 2014	Moderate	Y	Y	Y	U	U	U	Y	U	Y	Y	Y	Y	Y	Block randomization well described,

															but blinding and attrition handling incompletely detailed
Helfand et al., 2008	**Moderate**	Y	U	Y	U	U	U	Y	U	Y	Y	Y	Y	Y	Block randomization reported, but concealment/blinding incompletely described; follow-up losses present

**JBI Analytical Cross-Sectional Checklist Item-Level Risk of Bias**										
**Citation**	**Overall RoB**	**X1: Inclusion criteria clearly defined**	**X2: Subjects and setting described in detail**	**X3: Exposure measured validly and reliably**	**X4: Objective standard criteria used**	**X5: Confounding factors identified**	**X6: Strategies to deal with confounding stated**	**X7: Outcomes measured validly and reliably**	**X8: Appropriate statistical analysis**	**Key methodological concerns**
Kanga, 2021	**High**	Y	Y	Y	Y	N	N	Y	Y	Facility-based consecutive sampling; no confounder assessment; limited external validity
Díaz-Ortega et al., 2020	**Low**	Y	Y	Y	Y	Y	Y	Y	Y	Minor limitation inherent to cross-sectional design; otherwise methodologically strong
Hussain et al., 2013	**Moderate**	Y	Y	Y	Y	N	N	Y	Y	Non-randomized grouped comparison; serial/household recruitment; no confounder control
Hussain et al., 2024	**High**	U	Y	U	Y	N	N	Y	Y	Sampling method insufficiently described; vaccination verified by parental record; no multivariable control

**JBI Prevalence Checklist Item-Level Risk of Bias**											
**Citation**	**Overall RoB**	**P1: Sample frame appropriate**	**P2: Participants sampled appropriately**	**P3: Sample size adequate**	**P4: Subjects and setting described in detail**	**P5: Data analysis with sufficient coverage**	**P6: Valid methods for identifying condition**	**P7: Condition measured standard/reliable way**	**P8: Appropriate statistical analysis**	**P9: Response rate adequate or managed**	**Key methodological concerns**
Campbell et al., 2021	**Moderate**	Y	Y	Y	Y	U	Y	Y	Y	U	Good cluster sampling, but descriptive analysis only and mixed campaign/routine exposure
Prosperi et al., 2025	**Moderate**	Y	Y	Y	Y	U	Y	Y	Y	U	Campaign-participant sample rather than full population frame; limited adjustment

JBI Quasi-Experimental Checklist Item-Level Risk of Bias											
**Citation**	**Overall RoB**	**Q1: Cause and effect clear**	**Q2: Participants included in comparisons similar**	**Q3: Participants received similar care apart from intervention**	**Q4: Control group present**	**Q5: Multiple measurements pre/post**	**Q6: Follow-up complete; differences described/analysed**	**Q7: Outcomes measured same way**	**Q8: Outcomes measured reliably**	**Q9: Appropriate statistical analysis**	**Key methodological concerns**
Takechi et al., 2001	**High**	Y	U	U	N	Y	Y	Y	U	Y	No control group; campaign-attendee sample; assay reporting limited
Saffar et al., 2011	**Moderate**	Y	Y	U	N	Y	U	Y	Y	Y	No contemporary control group; blinding not reported
Karwi et al., 2020	**High**	Y	U	U	N	Y	U	Y	U	U	Reporting incomplete; no control group; limited statistical detail

Table S4. Full-text articles assessed for eligibility (n = 44), with final inclusion decision and reason

S. No.	Study	Title	Inclusion/Exclusion decision	Reason
**Included studies (n = 18)**				
1	(Tejiokem et al. 2025)	Pre- and Post-Vaccination Measles Antibody and Persistence Up to 5 Years of Age Among Early ART-Treated HIV-Infected, HIV-Exposed Uninfected and HIV-Unexposed Children in Cameroon	Included	Met review scope; relevant serological outcome in LMIC child population. Included in narrative/special-population synthesis.
2	(Eskenazi et al. 2025)	Undernutrition and antibody response to measles, tetanus and Haemophilus Influenzae type b (Hib) vaccination in pre-school South African children: The VHEMBE birth cohort study	Included	Met review scope: relevant measles antibody outcome in LMIC child population. Included in the determinants narrative synthesis.
3	(Campbell et al. 2021)	Linked vaccination coverage surveys plus serosurveys among Ethiopian toddlers undertaken three years apart to compare coverage and serologic evidence of protection in districts implementing the RED-QI approach	Included	Met review scope; relevant serosurvey evidence in LMIC children. Included in narrative/mixed-context synthesis.
4	(Takechi et al. 2001)	Measles sero-surveillance during mass immunisation campaign in Malawi	Included	Relevant measles serological evidence in LMIC children. Included in narrative/campaign-context synthesis.
5	(Kanga 2021)	Evaluation of Measles Immunity among Children Aged 9 to 59 Months at Selected Health Facilities in Kwale, Narok and Lamu Counties of Kenya	Included	Met review scope; post-vaccination measles serology in eligible LMIC child age range. Included in narrative serosurvey synthesis.
6	(Ibrahim et al. 2006)	Measles virus-specific antibody levels in Sudanese infants: a prospective study using filter-paper blood samples	Included	Relevant vaccine-related serological evidence in LMIC infants/young children. Included in narrative/timing-related synthesis.
7	(Sudfeld et al. 2013)	Effect of multivitamin supplementation on measles vaccine response among HIV-exposed uninfected Tanzanian infants	Included	Relevant measles vaccine serological outcome in LMIC special population. Included in determinants/special-population synthesis.
8	(Martins et al. 2014)	Measles Virus Antibody Responses in Children Randomly Assigned to Receive Standard-Titer Edmonston-Zagreb Measles Vaccine at 4.5 and 9 Months of Age, 9 Months of Age, or 9 and 18 Months of Age	Included	Relevant serological evidence but non-standard schedule; included in early high-risk/schedule-comparison narrative synthesis.
9	(Díaz-Ortega et al. 2020)	Seroprevalence of measles antibodies and factors associated with susceptibility: a national survey in Mexico using a plaque reduction neutralization test	Included	Relevant serological outcome with child subgroup data; included in narrative serosurvey/determinants synthesis.
10	(Prosperi et al. 2024)	High measles and rubella vaccine coverage and seroprevalence among Zambian children participating in a measles and rubella supplementary immunization activity	Included	Relevant serological evidence in LMIC children, but campaign-linked; included in mixed routine/campaign narrative synthesis.
11	(Saffar et al. 2011)	Measles–mumps–rubella revaccination; 18 months vs. 4–6 years of age: potential impacts of schedule changes	Included	Relevant serological comparison of second-dose timing; included in revaccination/schedule-comparison synthesis.
12	(Hussain et al. 2013)	Immune response to 1 and 2 dose regimens of measles vaccine in Pakistani children	Included	Directly eligible routine two-dose study for the primary review question; included in primary meta-analysis.
13	(Karwi, Hussein, and Wahid 2020)	Sero-conversion after Second Measles' Containing Vaccine in a Selected Sample of Young Children	Included	Relevant serological response after second dose in LMIC child population; included in narrative two-dose evidence.

14	(Verma et al. 2023)	Immunogenicity of measles-rubella vaccine administered under India's Universal Immunization Programme in the context of measles-rubella elimination goal: A longitudinal study	Included	Directly eligible routine two-dose study for the primary review question; included in primary meta-analysis.
15	(Martins et al. 2013)	Measles antibody levels after vaccination with Edmonston-Zagreb and Schwarz measles vaccine at 9 months or at 9 and 18 months of age: a serological study within a randomised trial of different measles vaccines	Included	Relevant serological study but schedule/strain comparison; included in revaccination/schedule-comparison narrative synthesis.
16	(Hussain et al. 2024)	Sero-surveillance of Measles Virus Seropositivity Amongst Vaccinated Children of Rural Areas of Sindh, Pakistan	Included	Relevant post-vaccination serosurvey in LMIC children; included in narrative serosurvey synthesis.
17	(Helfand et al. 2008)	Evaluation of the Immune Response to a 2-Dose Measles Vaccination Schedule Administered at 6 and 9 Months of Age to HIV-Infected and HIV-Uninfected Children in Malawi	Included	Relevant serological evidence but early high-risk/HIV-related schedule; included in special-population/early schedule synthesis.
18	(Fowlkes et al. 2011)	Persistence of Vaccine-Induced Measles Antibody Beyond Age 12 Months: A Comparison of Response to One and Two Doses of Edmonston-Zagreb Measles Vaccine Among HIV-Infected and Uninfected Children in Malawi	Included	Relevant persistence data in special-population context; included in special-population/persistence narrative synthesis.
	**Excluded full-text studies (n = 26)**			
1	(Rodriguez-Galet et al. 2025)	Immunity to six vaccine pathogens in HIV positive and HIV negative children in Equatorial Guinea	Excluded	Wrong outcome / not sufficiently focused on measles-specific post-vaccination seroprotection for the review question.
2	(Mburu et al. 2025)	Modelling the relative contribution of infection, routine vaccination and supplementary immunisation activities to measles seroconversion in Kenyan children	Excluded	Wrong design / modelling study; not a directly eligible primary serological study.
3	(Nielsen et al. 2022)	Effect of early two-dose measles vaccination on childhood mortality and modification by maternal measles antibody in Guinea-Bissau, West Africa: A single-centre open-label randomised controlled trial	Excluded	Wrong outcome; focused on mortality rather than vaccine-related serological protection outcome.
4	(Dixon et al. 2022)	Measles susceptibility in maternal-infant dyads, Bamako, Mali	Excluded	Wrong population / indirect population; maternal-infant dyad context not aligned with the review's vaccinated 1–5-year child focus.
5	(Semba et al. 1995)	Reduced seroconversion to measles in infants given vitamin A with measles vaccination	Excluded	Wrong study duration / publication outside prespecified time window.
6	(Benn et al. 1997)	Randomised trial of effect of vitamin A supplementation on antibody response to measles vaccine in Guinea-Bissau, West Africa	Excluded	Wrong study duration / publication outside prespecified time window.
7	(Halsey et al. 1985)	Response to measles vaccine in Haitian infants 6 to 12 months old. Influence of maternal antibodies, malnutrition, and concurrent illnesses	Excluded	Wrong study duration / publication outside prespecified time window.
8	(Waibale et al. 1999)	The effect of human immunodeficiency virus-1 infection and stunting on measles immunoglobulin-G levels in children vaccinated against measles in Uganda	Excluded	Wrong study duration / publication outside prespecified time window.

9	(Rosenthal and Clements 1993)	Two-dose measles vaccination schedules	Excluded	Background/older article; outside prespecified time window and not retained as an included primary study.
10	(Measles Vaccination in Guinea-Bissau. Strategies to Reduce Disease Burden and Improve Child Survival 2005)	Early Two-dose Measles Vaccination Trial	Excluded	Background article / not an included primary study for evidence synthesis.
11	(Quach et al. 2025)	Low Measles Seropositivity in Vaccinated Children	Excluded	Wrong population / not aligned with the review's target LMIC under-five population.
12	(Kohler et al. 2003)	Immunogenicity of Measles and Rubella Vaccines in Oman: A Prospective Clinical Trial	Excluded	Wrong population / setting not aligned with the review's target population criteria.
13	(Kumari and Kutty 2021)	Measles Specific Immunoglobulin G Response in Children Aged 4–12 Year Who Received Two Doses of Measles Containing Vaccine in Infancy	Excluded	Wrong population; age range extends beyond the review's target 1–5-year population.
14	(Sarawanangkoor et al. 2024)	Persistence of Antibodies against Measles, Mumps, and Rubella after the Two-Dose MMR Vaccination: A 7-Year Follow-Up Study	Excluded	Wrong population / long-term follow-up outside target child age range.
15	(Toyama et al. 2013)	Antibody against measles among first graders: Comparison of the antibody titer before and after the introduction of the two-dose measles-rubella vaccination program	Excluded	Wrong population; school-age children outside target age range.
16	(梁 et al., n.d.)	A Serological Effect Study on Measles Catch-up Mass Campaign	Excluded	Indirect campaign-only context / insufficient alignment with routine post-vaccination child seroprotection question.
17	(MMR-158 Study Group 2019)	A Second Dose of a Measles–Mumps–rubella Vaccine Administered to Healthy Four-to-six-year-old Children: a Phase III, Observer-Blind, Randomized, Safety and Immunogenicity Study Comparing GSK MMR and MMR II and with and without DTaP–IPV and Varicella Vaccines Co-Administration	Excluded	Wrong population; 4–6-year older child trial outside the intended LMIC routine under-five evidence framework.
18	(Marie Brønd 2018)	Randomized Trial of 2 Versus 1 Dose of Measles Vaccine: Effect on Hospital Admission of Children After 9 Months of Age	Excluded	Wrong outcome; hospital admission rather than serological protection outcome.
19	(J A Kaan, MD MedMicrobiol and P C A van Vlokhoven, MD n.d.)	Immunogenicity of measles vaccine from a hospital-based and outreach programme in rural Kenya	Excluded	Wrong study duration / publication outside prespecified time window.
20	(Aaby et al. 1989)	Child mortality related to seroconversion or lack of seroconversion after measles vaccination	Excluded	Wrong outcome; mortality-focused rather than primary seroprotection outcome.
21	(Hh et al. 2020)	Sero-Prevalence of Measles in Southern Darfur State, Sudan	Excluded	Wrong population / unclear vaccinated under-five serological focus relative to review question.

22	(Haranalli et al. 2025)	Seroprotection against hepatitis B, measles, mumps, and rubella in children with diabetes	Excluded	Wrong population; special clinical group outside the review's intended vaccinated child population framework.
23	(Kumar et al. 2023)	Evaluating effective measles vaccine coverage in the Malaysian population accounting for between-dose correlation and vaccine efficacy	Excluded	Wrong population and wrong design; coverage/model-based analysis without primary serological outcome.
24	(Memon M et al. 2025)	Coverage, timeliness of measles immunisation and its predictors in Pakistan: an analysis of 6.2 million children enrolled in the Provincial Electronic Immunisation Registry	Excluded	Wrong outcome; vaccination coverage/timeliness study without serological outcome.
25	(Mahazabin et al. 2024)	Factors influencing the first dose of measles vaccination uptake in 42 low- and middle-income countries	Excluded	Wrong outcome; uptake/coverage determinants without serological endpoint.
26	(Nurgi et al. 2024)	Determinants of measles second dose vaccination dropout among children aged 18–24 months in , central Ethiopia; unmatched case-control study	Excluded	Wrong outcome; dropout determinants without serological outcome.

Table S5. Summary of laboratory methods, seroprotection thresholds, and methodological heterogeneity across included studies

Study	Study design	Laboratory assay	Seroprotection threshold/definition	Timing of antibody assessment	Principal source of heterogeneity
Tejiokem et al., 2025	Prospective cohort	ELISA	300–335 mIU/mL	Birth–60 months	HIV-specific cohort; long-term follow-up
Eskenazi et al., 2025	Prospective cohort	ELISA	250 mIU/mL	3.5–5 years	Long-term antibody persistence
Campbell et al., 2021	Repeated cross-sectional	PRNT	≥120 mIU/mL	Cross-sectional	Mixed routine and SIA exposure
Takechi et al., 2001	Cross-sectional	Particle agglutination	Assay-specific / not reported	Baseline and 8 weeks	Campaign population; obsolete assay
Kanga, 2021	Cross-sectional	ELISA + PRNT	200 mIU/mL	Cross-sectional	Facility-based sampling
Ibrahim et al., 2006	Prospective cohort	Measles virus-specific IgG ELISA + F/H-specific IgG immunofluorescence assay	≥0.2 IU/mL measles virus-specific IgG by ELISA	Birth–24 months	Maternal antibody study
Sudfeld et al., 2013	Randomized trial	ELISA	200 mIU/mL	15–18 months	HIV-exposed infants only
Martins et al., 2013	Randomized trial	Hemagglutination inhibition	125 mIU/mL	4.5–24 months	Early vaccination schedule comparison
Díaz-Ortega et al., 2020	National serosurvey	PRNT	≥120 mIU/mL	Cross-sectional	National population survey
Prosperi et al., 2025	Cross-sectional	Multiplex bead assay (EIA calibrated)	153 mIU/mL	During SIA	Campaign-based population
Saffar et al., 2011	Comparative observational	ELISA	275 mIU/mL	Pre/post MMR2	Revaccination study
Hussain et al., 2013	Comparative observational	ELISA	300–335 mIU/mL	8–11 weeks and 5 years	Mixed short- and long-term follow-up
Karwi et al., 2020	Pre/post observational	ELISA	Assay-specific / not reported	4–12 weeks	Small clinic-based sample
Verma et al., 2023	Prospective cohort	ELISA	>10 IU/mL	4–6 weeks	Early post-vaccination immunogenicity
Martins et al., 2014	Randomized trial	Hemagglutination inhibition	125 mIU/mL	18–24 months	Vaccine-strain comparison
Hussain et al., 2024	Cross-sectional	ELISA	Assay-specific / not reported	Single survey	Rural community survey
Helfand et al., 2008	Randomized trial	EIA	Assay-specific / not reported	3 months	HIV-focused schedule trial
Fowlkes et al., 2011	Longitudinal cohort	EIA + PRNT	≥120 mIU/mL	24 months	Long-term antibody persistence

Note: Seroprotection thresholds and definitions are reported exactly as specified in the original studies and were not harmonized across assays. Numerical thresholds ranged across studies, while some studies used assay-specific definitions or did not report a numerical cutoff. Differences in assay platform, calibration, antibody measurement, and timing of assessment limit direct comparison across studies.

Table S6. Study-level determinants of measles serological protection across included studies

Study	Main determinant(s) identified	Direction of association	Interpretation
Tejiokem et al., 2025	HIV status; completed multi-dose schedule; time since vaccination	HIV-infected children had lower baseline antibody positivity; protection remained high after completing the schedule	Complete schedules may reduce HIV-related differences in post-vaccination protection, although titres wane over time.
Eskenazi et al., 2025	Stunting; undernutrition	Lower antibody concentrations	Undernutrition was associated with reduced measles antibody persistence.
Campbell et al., 2021	District programme performance; SIA exposure	Variable improvement by district	Seroprotection reflected programme quality and local immunization performance.
Takechi et al., 2001	Age; campaign vaccination; previous vaccination	Higher protection with increasing age and campaign dose	Campaign vaccination boosted titres and closed immunity gaps among susceptible children.
Kanga, 2021	Vaccination status; documentation; sex	Vaccination strongly associated; sex not associated	Verified vaccination history was a key predictor of seroprotection.
Ibrahim et al., 2006	Maternal antibody decay; infant age	Maternal antibodies declined rapidly by 6 months	Findings supported routine measles vaccination at 9 months.
Sudfeld et al., 2013	Multivitamin supplementation; nutrition; maternal HIV severity; age at vaccination	Supplementation had no benefit; poor nutrition and maternal HIV severity reduced response	Nutritional and maternal factors influenced immune response more than supplementation.
Martins et al., 2014	Age at first dose; number of doses	Two-dose schedules gave higher protection	Early vaccination was immunogenic, but completion of two doses was essential.
Díaz-Ortega et al., 2020	Household crowding; unknown vaccination status; age	Increased susceptibility	Susceptibility clustered in children with social and programmatic risk factors.
Prosperi et al., 2025	Prior MR doses; SIA participation; household structure	Prior doses increased seroprevalence	Routine dose history and campaign reach shaped population immunity.
Saffar et al., 2011	Timing of second MMR dose	Higher protection after revaccination	Second-dose timing improved immunity in both younger and older children.

Hussain et al., 2013	One-dose vs two-dose schedule; time since vaccination	Two doses higher; one-dose protection waned	Two-dose vaccination produced stronger and more durable protection.
Karwi et al., 2020	Receipt of second measles-containing vaccine	Increased seroprotection	The second dose substantially improved serological protection.
Verma et al., 2023	MR1 vs MR2	Higher protection after MR2	Completion of MR2 achieved near-complete seroprotection.
Martins et al., 2013	Vaccine strain; one vs two doses	Higher titres after two doses	Booster dosing improved antibody levels across vaccine strains.
Hussain et al., 2024	Rural setting; vaccination documentation; time since vaccination	Low protection despite reported vaccination	Reported vaccination alone did not ensure measurable immunity in rural Pakistan.
Helfand et al., 2008	HIV infection; early two-dose schedule	HIV reduced response; two doses improved protection in HIV-uninfected children	HIV infection remained a major biological barrier to vaccine response.
Fowlkes et al., 2011	HIV infection; antibody persistence; dose schedule	HIV reduced persistence; HIV-uninfected children maintained protection	Long-term persistence was poorest among HIV-infected children.

Table S7. Certainty of evidence for serological protection outcomes following routine two-dose measles vaccination in children 1–5 years in LMICs – Using GRADE

Outcome	No. studies (No. participants)	Summary of findings (seroprotection)	Certainty (GRADE) and reasons
**Seroprotection after two routine MCV doses** (primary)	18 (≈23,236) [64†L99-L107]	High: Most studies reported ≥~80–95% seroprotection after two doses [64†L99-L107].	Low: All evidence is observational. Downgraded for risk of bias (many studies had limitations in design or sampling) and inconsistency (very high heterogeneity in reported seroprotection [64†L99-L107]). Imprecision is moderate (wide CIs in small studies). No upgrade was applied.
**Seroprotection by time since vaccination**	Subsets of above (data not pooled)	Generally stable in early years but waning over time. For example, seroprotection was ~93% within 10y post-vaccination vs ~83% at 16–22y【64†L109-L112】.	Low: Observational data. Some consistency in trend (decline with time【64†L109-L112】) but wide CIs for longer intervals (imprecision). Downgraded for imprecision (small numbers in long-term strata) and some risk of bias (heterogeneous assays/sampling across studies).
**Seroprotection by age at first dose (MCV1)**	Few (≈5)	Very high overall (>95%) after two doses, regardless of first-dose age. Seroconversion after 2 doses was ~98% even if MCV1 was given before 9 months【54†L179-L187】. Slight (non-significant) reduction (~4%) was reported for children with very early MCV1【54†L179-L187】.	Low: Observational. Data suggest uniformly high protection, with possibly small differences by age. Downgraded one level for risk of bias (few controlled comparisons, confounding by country/schedule) but *not* for inconsistency (findings were largely consistent). Overall certainty is low.
**Seroprotection by HIV status**	~5	Lower in HIV-infected. Published meta-analyses show HIV+ children often have substantially reduced seroconversion (e.g., ~68% seroprotected in HIV+ vs ~95% in uninfected)【64†L99-L107】.	Low: Observational (small cohorts). Consistently lower immunity in HIV-infected (large effect size). Downgraded for imprecision (small sample sizes, wide CIs in subgroups) and risk of bias (selection bias, treatment status variability). The consistency of the effect (HIV always worse) offsets one downgrade, but we keep evidence at low.
**Seroprotection by nutritional status**	3–4	Some evidence suggests undernourished children have poorer immune response. (Reviews note measles vaccine often yields lower seroprotection and efficacy in malnourished children【5†】) In our included studies, results were sparse and varied.	Very low: Observational and indirect. Few studies reported nutrition data; definitions varied. Downgraded for indirectness (nutrition not always clearly measured), inconsistency (mixed findings), and imprecision (small samples). Even with a plausible effect (Prendergast 2015), certainty is very low.
**Seroprotection by assay type**	18 (multiple assays)	Variable. Different assays and cut-offs were used (e.g. PRNT vs ELISA, 120–200 mIU/mL thresholds), so reported seroprotection was not directly comparable. Generally, PRNT tends to give higher antibody titers, affecting estimates.	Very low: All data indirect comparisons. The outcome is methodological: assays varied across studies. Downgraded for inconsistency (systematic methodological heterogeneity) and indirectness (outcome depends on lab method). Certainty is very low given the lack of standardized measures.

Table S8. Evidence Gaps identified across the included studies

Domain	Evidence gap identified	Evidence from included studies	Potential implication
Geographic representation	Most studies originated from sub-Saharan Africa, with limited evidence from Asia and Latin America	10 of 18 studies were conducted in AFRO; few nationally representative studies from other LMIC regions	Limited generalizability across LMICs
Long-term antibody persistence	Few studies evaluated immunity beyond two years after completion of routine vaccination	Most studies measured antibodies within weeks or months after vaccination; only a small number assessed persistence to 3–5 years	Limited understanding of long-term protection and waning immunity
Standardization of seroprotection thresholds	Wide variation in antibody cut-offs (120–335 mIU/mL and assay-specific thresholds)	Different studies used different laboratory definitions of protection	Direct comparison of seroprotection estimates is difficult
Laboratory methods	Multiple serological assays were used (ELISA, PRNT, EIA, HI, particle agglutination, multiplex assays)	Assay sensitivity and specificity varied substantially	Methodological heterogeneity limits comparability
Vaccination verification	Vaccination status was inconsistently verified	Some studies used immunization cards or clinic records, whereas others relied on parental recall or unclear methods	Increased potential for exposure misclassification
Follow-up duration	Considerable variation in timing of antibody assessment	Assessments ranged from 4 weeks after vaccination to 5 years post-vaccination	Difficult to distinguish primary response from long-term persistence
Population representativeness	Several studies were hospital-, clinic-, or campaign-based	Limited number of nationally representative serosurveys	Reduced external validity
Determinants of immunity	Important determinants were inconsistently evaluated	HIV status and nutritional status were frequently investigated, whereas socioeconomic, maternal, and health-system factors were rarely assessed	Limited understanding of drivers of immunity gaps
Statistical analysis	Few studies adjusted for confounding	Most analyses were descriptive or univariable	Reduced certainty regarding causal determinants
Reporting quality	Incomplete reporting of laboratory protocols, thresholds, vaccination history, and follow-up	Several studies lacked detailed methodological information	Reduced reproducibility and evidence synthesis

References:

1. Aaby, P., I. R. Pedersen, K. Knudsen, M. C. da Silva, C. H. Mordhorst, N. C. Helm-Petersen, B. S. Hansen, J. Thårup, A. Poulsen, and M. Sodemann. 1989. “Child Mortality Related to Seroconversion or Lack of Seroconversion after Measles Vaccination.” *The Pediatric Infectious Disease Journal* 8(4):197–200.
2. Benn, C. S., P. Aaby, C. Balé, J. Olsen, K. F. Michaelsen, E. George, and H. Whittle. 1997. “Randomised Trial of Effect of Vitamin A Supplementation on Antibody Response to Measles Vaccine in Guinea-Bissau, West Africa.” *Lancet (London, England)* 350(9071):101–5. doi:10.1016/S0140-6736(96)12019-5.
3. Campbell, James D., Marcela F. Pasetti, Lisa Oot, Zenaw Adam, Mesfin Tefera, Berhane Beyane, Nigisti Mulholland, Robert Steinglass, Rebecca Krey, Wilbur H. Chen, William C. Blackwelder, and Myron M. Levine. 2021. “Linked Vaccination Coverage Surveys plus Serosurveys among Ethiopian Toddlers Undertaken Three Years Apart to Compare Coverage and Serologic Evidence of Protection in Districts Implementing the RED-QI Approach.” *Vaccine* 39(40):5802–13. doi:10.1016/j.vaccine.2021.08.071.
4. Díaz-Ortega, José Luis, Elizabeth Ferreira-Guerrero, Luis Pablo Cruz-Hervert, Guadalupe Delgado-Sánchez, Leticia Ferreyra-Reyes, Mercedes Yanes-Lane, Norma Mongua-Rodríguez, Rogelio Montero-Campos, Deyanira Castañeda-Desales, and Lourdes García-García. 2020. “Seroprevalence of Measles Antibodies and Factors Associated with Susceptibility: A National Survey in Mexico Using a Plaque Reduction Neutralization Test.” *Scientific Reports* 10(1):17488. doi:10.1038/s41598-020-73618-8.
5. Dixon, Meredith G., Milagritos D. Tapia, Kathleen Wannemuehler, Richard Luce, Mark Papania, Samba Sow, Myron M. Levine, and Marcela F. Pasetti. 2022. “Measles Susceptibility in Maternal-Infant Dyads-Bamako, Mali.” *Vaccine* 40(9):1316–22. doi:10.1016/j.vaccine.2022.01.012.
6. Eskenazi, Brenda, Stephen Rauch, Basant Elsiwi, Riana Bornman, Muvhulawa Obida, Angela Brewer, Brian J. Ward, and Jonathan Chevrier. 2025. “Undernutrition and Antibody Response to Measles, Tetanus and Haemophilus Influenzae Type b (Hib) Vaccination in Pre-School South African Children: The VHEMBE Birth Cohort Study.” *Vaccine* 46:126564. doi:10.1016/j.vaccine.2024.126564.
7. Fowlkes, Ashley, Desiree Witte, Judy Beeler, Susette Audet, Philip Garcia, Aaron Curns, Chunfu Yang, Richard Fudzulani, Robin Broadhead, William J. Bellini, Felicity Cutts, and Rita F. Helfand. 2011. “Persistence of Vaccine-Induced Measles Antibody Beyond Age 12 Months: A Comparison of Response to One and Two Doses of Edmonston-Zagreb Measles Vaccine Among HIV-Infected and Uninfected Children in Malawi.” *The Journal of Infectious Diseases* 204(suppl_1):S149–57. doi:10.1093/infdis/jir135.
8. Halsey, N. A., R. Boulos, F. Mode, J. Andre, L. Bowman, R. G. Yaeger, S. Toureau, J. Rohde, and C. Boulos. 1985. “Response to Measles Vaccine in Haitian Infants 6 to 12 Months Old. Influence of Maternal Antibodies, Malnutrition, and Concurrent Illnesses.” *The New England Journal of Medicine* 313(9):544–49. doi:10.1056/NEJM198508293130904.
9. Haranalli, Maruti, Sangeeta Yadav, Aashima Dabas, and Vikas Manchanda. 2025. “Seroprotection against Hepatitis B, Measles, Mumps, and Rubella in Children with Diabetes.” *Journal of Tropical Pediatrics* 71(4):fmaf026. doi:10.1093/tropej/fmaf026.

10. Helfand, Rita F., Desiree Witte, Ashley Fowlkes, Philip Garcia, Chunfu Yang, Richard Fudzulani, Laura Walls, Sun Bae, Peter Strebel, Robin Broadhead, William J. Bellini, and Felicity Cutts. 2008. “Evaluation of the Immune Response to a 2-Dose Measles Vaccination Schedule Administered at 6 and 9 Months of Age to HIV-Infected and HIV-Uninfected Children in Malawi.” *The Journal of Infectious Diseases* 198(10):1457–65. doi:10.1086/592756.
11. Hh, Musa, Musa Th, Kambo Ri, Ahmed Aa, and Musa Ih. 2020. “Sero-Prevalence of Measles in Southern Darfur State, Sudan.” *Journal of Emerging Diseases and Virology* 5(2). doi:10.16966/2473-1846.154.
12. Hussain, Hamidah, Dure Samin Akram, Subhash Chandir, Aamir J. Khan, Ashraf Memon, and Neal A. Halsey. 2013. “Immune Response to 1 and 2 Dose Regimens of Measles Vaccine in Pakistani Children.” *Human Vaccines & Immunotherapeutics* 9(12):2529–32. doi:10.4161/hv.25993.
13. Hussain, Zeb, Ambreen Fatima, Muhammad Sameer Qureshi, Asif Iqbal Khan, and Shaheen Sharafat. 2024. “Sero-Surveillance of Measles Virus Seropositivity Amongst Vaccinated Children of Rural Areas of Sindh, Pakistan.” *Microbiological & Immunological Communications* 3(01):73–80. doi:10.55627/mic.003.001.0513.
14. Ibrahim, S. A., A. Abdallah, E. A. Saleh, A. D. M. E. Osterhaus, and R. L. De Swart. 2006. “Measles Virus-Specific Antibody Levels in Sudanese Infants: A Prospective Study Using Filter-Paper Blood Samples.” *Epidemiology and Infection* 134(1):79–85. doi:10.1017/S0950268805004620.
15. J A Kaan, MD MedMicrobiol, and P C A van Vlokhoven, MD. n.d. “Immunogenicity of Measles Vaccine from a Hospital Based and Outreach Programme in Rural Kenya - J A Kaan, P C A van Vlokhoven, P M Schneeberger, W Nijhof, 1992.” Retrieved July 10, 2026. https://journals.sagepub.com/doi/10.1177/004947559202200110?url_ver=Z39.88-2003&rfr_id=ori:rid:crossref.org&rfr_dat=cr_pub%20%200pubmed.
16. Kanga, Ali Juma. 2021. “Evaluation of Measles Immunity among Children Aged 9 to 59 Months at Selected Health Facilities in Kwale, Narok and Lamu Counties of Kenya.” PhD Thesis, JKUAT-COHES.
17. Karwi, Yahya G., Reem G. Hussein, and Salwa Sh. Abdul- Wahid. 2020. “Sero-Conversion after Second Measles’ Containing Vaccine in a Selected Sample of Young Children.” *Journal of Pharmaceutical Research International* 20–27. doi:10.9734/jpri/2020/v32i2730851.
18. Kohler, Kathryn A., Ali Jaffer M. Suleiman, Susan E. Robertson, Pradeep Malankar, Saleh Al-Khusaiby, Rita F. Helfand, David Brown, William J. Bellini, and Roland W. Sutter. 2003. “Immunogenicity of Measles and Rubella Vaccines in Oman: A Prospective Clinical Trial.” *The Journal of Infectious Diseases* 187(s1):S177–85. doi:10.1086/368048.
19. Kumar, Shurendar Selva, Anna-Maria Hartner, Arunah Chandran, Katy A. M. Gaythorpe, and Xiang Li. 2023. “Evaluating Effective Measles Vaccine Coverage in the Malaysian Population Accounting for Between-Dose Correlation and Vaccine Efficacy.” *BMC Public Health* 23(1):2351. doi:10.1186/s12889-023-17082-9.
20. Kumari, P. Leela, and Alka Madhavan Kutty. 2021. “Measles Specific Immunoglobulin G Response in Children Aged 4-12 Year Who Received Two Doses of Measles Containing Vaccine in Infancy.” *Indian Pediatrics* 58(3):250–52.

21. Mahazabin, Maliha, Shangita Nandi Swarna, Jannatul Naime, Abdur Rahman, and Md Akhtarul Islam. 2024. “Factors Influencing the First Dose of Measles Vaccination Uptake in 42 Low- and Middle-Income Countries.” *Public Health* 237:85–93. doi:10.1016/j.puhe.2024.09.017.
22. Marie Brønd. 2018. “Randomized Trial of 2 Versus 1 Dose of Measles Vaccine: Effect on Hospital Admission of Children After 9 Months of Age - PubMed.” https://pubmed.ncbi.nlm.nih.gov/28992261/.
23. Martins, C., M. L. Garly, C. Bale, A. Rodrigues, J. Njie-Jobe, C. S. Benn, H. Whittle, and P. Aaby. 2014. “Measles Virus Antibody Responses in Children Randomly Assigned to Receive Standard-Titer Edmonston-Zagreb Measles Vaccine at 4.5 and 9 Months of Age, 9 Months of Age, or 9 and 18 Months of Age.” *Journal of Infectious Diseases* 210(5):693–700. doi:10.1093/infdis/jiu117.
24. Martins, Cesario, May-Lill Garly, Carlitos Bale, Amabelia Rodrigues, Christine S. Benn, Hilton Whittle, and Peter Aaby. 2013. “Measles Antibody Levels after Vaccination with Edmonston-Zagreb and Schwarz Measles Vaccine at 9 Months or at 9 and 18 Months of Age: A Serological Study within a Randomised Trial of Different Measles Vaccines.” *Vaccine* 31(48):5766–71. doi:10.1016/j.vaccine.2013.08.044.
25. Mburu, Caroline, John Ojal, Rose Selim, Rose Ombati, Donald Akech, Boniface Karia, James Tuju, Antipa Sigilai, Gaby Smits, Pieter van Gageldonk, Fiona van der Klis, Eunice Kagucia, Anthony Scott, Ifedayo Adetifa, and Stefan Flasche. 2025. “Modelling the Relative Contribution of Infection, Routine Vaccination and Supplementary Immunisation Activities to Measles Seroconversion in Kenyan Children.” *PLoS Computational Biology* 21(9):e1013531. doi:10.1371/journal.pcbi.1013531.
26. Measles Vaccination in Guinea-Bissau. Strategies to Reduce Disease Burden and Improve Child Survival. 2005. https://clinicaltrials.gov/study/NCT00168558.
27. Memon M, Siddiqi DA, Dharma VK, Shah MT, Iftikhar S, Setayesh H, and Chandir S. 2025. “Coverage, Timeliness of Measles Immunisation and Its Predictors in Pakistan: An Analysis of 6.2 Million Children Enrolled in the Provincial Electronic Immunisation Registry.” *BMJ Glob Health* 10(3). doi:10.1136/bmjgh-2024-016717.
28. MMR-158 Study Group. 2019. “A Second Dose of a Measles-Mumps-Rubella Vaccine Administered to Healthy Four-to-Six-Year-Old Children: A Phase III, Observer-Blind, Randomized, Safety and Immunogenicity Study Comparing GSK MMR and MMR II with and without DTaP-IPV and Varicella Vaccines Co-Administration.” *Human Vaccines & Immunotherapeutics* 15(4):786–99. doi:10.1080/21645515.2018.1554971.
29. Nielsen, Sebastian, Ane B. Fisker, Isaquel da Silva, Stine Byberg, Sofie Biering-Sørensen, Carlitos Balé, Amarildo Barbosa, Morten Bjerregaard-Andersen, Nadja Skadkær Hansen, Vu An Do, Ole Bæk, Stine Møller Rasmussen, Lone Damkjær, Sophus Hvidt, Olga Baltzersen, Amabelia Rodrigues, Cesario Martins, Kristoffer J. Jensen, Hilton C. Whittle, Gaby Smits, Fiona van der Klis, Peter Aaby, and Christine S. Benn. 2022. “Effect of Early Two-Dose Measles Vaccination on Childhood Mortality and Modification by Maternal Measles Antibody in Guinea-Bissau, West Africa: A Single-Centre Open-Label Randomised Controlled Trial.” *EClinicalMedicine* 49:101467. doi:10.1016/j.eclinm.2022.101467.

30. Nurgi, Kitessa, Seifadin Ahmed, Gemechu Ganfure, and Gemechu Gelan Bekele. 2024. "Determinants of Measles Second Dose Vaccination Dropout among Children Aged 18–24 Months in Ejere Woreda, Central Ethiopia; Unmatched Case-Control Study." *Frontiers in Pediatrics* 12:1432762. doi:10.3389/fped.2024.1432762.
31. Prosperi, Christine, Shaun A. Truelove, Andrea C. Carcelen, Gershom Chongwe, Francis D. Mwansa, Phillimon Ndubani, Edgar Simulundu, Innocent C. Bwalya, Mutinta Hamahuwa, Kelvin Kapungu, Kalumbu H. Matakala, Gloria Musukwa, Irene Mutale, Evans Betha, Nchimunya Chaavwa, Lombe Kampamba, Japhet Matoba, Passwell Munachoonga, Webster Mufwambi, Ken Situtu, Phillip E. Thuma, Constance Sakala, Princess Kayeye, Amy K. Winter, Matthew J. Ferrari, William J. Moss, and Simon Mutembo. 2024. "High Measles and Rubella Vaccine Coverage and Seroprevalence among Zambian Children Participating in a Measles and Rubella Supplementary Immunization Activity."
32. Quach, Huy Quang, Sara P. Jones, Iype Joseph, Alexandria J. Powell, Inna G. Ovsyannikova, Nathaniel D. Warner, Diane E. Grill, John B. Johnson, Remya Vasanthi Sasi, Archana Mohankumar Ajithakumari, Raji Prasad, Remya Reveendran, Jayalekshmi Devakikutty, Vishnu Vikraman Mohanakumari, Gregory A. Poland, M. Radhakrishna Pillai, Joshy Jacob, and Richard B. Kennedy. 2025. "Low Measles Seropositivity in Vaccinated Children." *JAMA Network Open* 8(8):e2529409. doi:10.1001/jamanetworkopen.2025.29409.
33. Rodriguez-Galet, Ana, Judit Ventosa-Cubillo, Manuel Eyene, Teresa Mikue-Owono, Jesús Nzang, Policarpo Ncogo, Agustín Benito, Angela Domínguez-García, and África Holguín. 2025. "Immunity to Six Vaccine Pathogens in HIV Positive and HIV Negative Children in Equatorial Guinea." *Scientific Reports* 15(1):45747. doi:10.1038/s41598-025-28643-w.
34. Rosenthal, S. R., and C. J. Clements. 1993. "Two-Dose Measles Vaccination Schedules." *Bulletin of the World Health Organization* 71(3–4):421–28.
35. Saffar, M. J., G. R. Fathpour, M. R. Parsaei, A. Ajami, A. R. Khalilian, J. Shojaei, and H. Saffar. 2011. "Measles-Mumps-Rubella Revaccination; 18 Months vs. 4-6 Years of Age: Potential Impacts of Schedule Changes." *Journal of Tropical Pediatrics* 57(5):347–51. doi:10.1093/tropej/fmq102.
36. Sarawanangkoor, Nasiri, Nasamon Wanlapakorn, Donchida Srimuan, Thaksaporn Thatsanathorn, Thanunrat Thongmee, and Yong Poovorawan. 2024. "Persistence of Antibodies against Measles, Mumps, and Rubella after the Two-Dose MMR Vaccination: A 7-Year Follow-Up Study." *Vaccines* 12(7):744. doi:10.3390/vaccines12070744.
37. Semba, R. D., Z. Munasir, J. Beeler, A. Akib, Muhilal, S. Audet, and A. Sommer. 1995. "Reduced Seroconversion to Measles in Infants given Vitamin A with Measles Vaccination." *Lancet (London, England)* 345(8961):1330–32. doi:10.1016/s0140-6736(95)92536-8.
38. Sudfeld, Christopher R., Christopher Duggan, Alex Histed, Karim P. Manji, Simin N. Meydani, Said Aboud, Molin Wang, Edward L. Giovannucci, and Wafaie W. Fawzi. 2013. "Effect of Multivitamin Supplementation on Measles Vaccine Response among HIV-Exposed Uninfected Tanzanian Infants." *Clinical and Vaccine Immunology* 20(8):1123–32. doi:10.1128/CVI.00183-13.
39. Takechi, M., M. Matsuo, D. Butao, I. L. Zungu, I. Chakanika, and J. Michongwe. 2001. "Measles Sero-Surveillance during Mass Immunisation Campaign in Malawi." *East African Medical Journal* 78(1):4–8. doi:10.4314/eamj.v78i1.9103.

40. Tejiokem, Mathurin Cyrille, Emilie Desselas, Thierry Joel Noumsi, Francis Ateba Ndongo, Suzie Tetang Ndiang, Mireille Arlette Fossi, Georgette Guemkam, Berenice Zangue Kenfack Tekougang, Paul Alain Tagnouokam-Ngoupo, Ida Calixte Penda, Albert Faye, and Josiane Warszawski. 2025. "Pre- and Post-Vaccination Measles Antibody and Persistence Up to 5 Years of Age Among Early ART-Treated HIV-Infected, HIV-Exposed Uninfected and HIV-Unexposed Children in Cameroon." *Vaccines* 13(6):584. doi:10.3390/vaccines13060584.
41. Toyama, Chisuzu, Seiichiro Nanri, Mitsuaki Tokumura, Mikako Inokuchi, Shioko Kawai, Tetsuya Tanaka, Yuko Tanaka, and Tetsuo Nakayama. 2013. "Antibody against Measles among First Graders: Comparison of the Antibody Titer before and after the Introduction of the Two-Dose Measles-Rubella Vaccination Program."
42. Verma, Santosh Kumar, Dheeraj Shah, Aaradhana Singh, Praveen Kumar Singh, Shukla Das, and Piyush Gupta. 2023. "Immunogenicity of Measles-Rubella Vaccine Administered under India's Universal Immunization Programme in the Context of Measles–Rubella Elimination Goal: A Longitudinal Study." *Indian Journal of Medical Research* 157(4):250–58. doi:10.4103/ijmr.IJMR_4113_20.
43. Waibale, P., S. J. Bowlin, E. A. Jr Mortimer, and C. Whalen. 1999. "The Effect of Human Immunodeficiency Virus-1 Infection and Stunting on Measles Immunoglobulin-G Levels in Children Vaccinated against Measles in Uganda." *International Journal of Epidemiology* 28(2):341–46. doi:10.1093/ije/28.2.341.
